# Repeated measurements of Adaptive Force: Maximal holding capacity differs from other maximal strength parameters and preliminary characteristics for non-professional strength vs. endurance athletes

**DOI:** 10.3389/fphys.2023.1020954

**Published:** 2023-02-22

**Authors:** Laura V. Schaefer, Friederike Carnarius, Silas Dech, Frank N. Bittmann

**Affiliations:** Neuromechanics Laboratory, Regulative Physiology and Prevention, Department Sports and Health Sciences, University Potsdam, Potsdam, Germany

**Keywords:** Adaptive Force, maximal isometric Adaptive Force, holding capacity, neuromuscular control, strength vs. endurance athletes, injury mechanisms, repeated adaptive isometric–eccentric muscle action, holding (HIMA) and pushing (PIMA) isometric muscle action

## Abstract

The Adaptive Force (AF) reflects the neuromuscular capacity to adapt to external loads during holding muscle actions and is similar to motions in real life and sports. The maximal isometric AF (AFiso_max_) was considered to be the most relevant parameter and was assumed to have major importance regarding injury mechanisms and the development of musculoskeletal pain. The aim of this study was to investigate the behavior of different torque parameters over the course of 30 repeated maximal AF trials. In addition, maximal holding vs. maximal pushing isometric muscle actions were compared. A side consideration was the behavior of torques in the course of repeated AF actions when comparing strength and endurance athletes. The elbow flexors of *n* = 12 males (six strength/six endurance athletes, non-professionals) were measured 30 times (120 s rest) using a pneumatic device. Maximal voluntary isometric contraction (MVIC) was measured pre and post. MVIC, AFiso_max_, and AF_max_ (maximal torque of one AF measurement) were evaluated regarding different considerations and statistical tests. AF_max_ and AFiso_max_ declined in the course of 30 trials [slope regression (mean ± standard deviation): AF_max_ = −0.323 ± 0.263; AFiso_max_ = −0.45 ± 0.45]. The decline from start to end amounted to −12.8% ± 8.3% (*p* < 0.001) for AF_max_ and −25.41% ± 26.40% (*p* < 0.001) for AFiso_max_. AF parameters declined more in strength vs. endurance athletes. Thereby, strength athletes showed a rather stable decline for AF_max_ and a plateau formation for AFiso_max_ after 15 trials. In contrast, endurance athletes reduced their AF_max_, especially after the first five trials, and remained on a rather similar level for AFiso_max_. The maximum of AFiso_max_ of all 30 trials amounted 67.67% ± 13.60% of MVIC (*p* < 0.001, *n* = 12), supporting the hypothesis of two types of isometric muscle action (holding vs. pushing). The findings provided the first data on the behavior of torque parameters after repeated isometric–eccentric actions and revealed further insights into neuromuscular control strategies. Additionally, they highlight the importance of investigating AF parameters in athletes based on the different behaviors compared to MVIC. This is assumed to be especially relevant regarding injury mechanisms.

## 1 Introduction

In daily activities and sports, the neuromuscular system has to frequently react and adapt to external forces. However, the adaptation of the neuromuscular system to external loads is usually not investigated in sports or movement sciences. Strength is commonly measured by pushing against resistance without considering the adaptive component. The Adaptive Force (AF) describes the neuromuscular capacity to adapt to externally varying forces, especially in an isometric holding manner (Hoff et al., 2015; Schaefer et al., 2017; Schaefer and Bittmann, 2019; Bittmann et al., 2020; Dech et al., 2021; Schaefer et al., 2021a; Schaefer et al., 2021b; Schaefer et al., 2022b). During holding activities, the neuromuscular system needs to adapt to external forces, which was assumed to require higher control and regulation processes than during pushing actions (Schaefer and Bittmann, 2017; Schaefer et al., 2021a; Schaefer et al., 2021b; Schaefer and Bittmann, 2021; Schaefer et al., 2022b; Schaefer and Bittmann, 2023). It was suggested that two types of isometric muscle action exist: the holding and the pushing isometric muscle action (HIMA and PIMA, respectively) (Schaefer and Bittmann, 2017; Schaefer and Bittmann, 2021; Schaefer and Bittmann, 2023). This is based on different studies indicating that, *inter alia*, a constant force can be maintained significantly longer in an isometric position during PIMA than during HIMA (Hunter et al., 2002; Rudroff et al., 2007; Rudroff et al., 2011; Rudroff et al., 2013; Schaefer and Bittmann, 2017; Schaefer and Bittmann, 2021). The AF is based on HIMA, whereby the participant has the task of maintaining an isometric position despite an increasing external load. Thus, the muscle tension has to be adapted to the external load, whereby the muscle length should stay stable. If the maximal holding capacity (maximal isometric AF; AFiso_max_) is exceeded, the participant merges into eccentric muscle action, whereby the force usually rises further until the maximal AF (AF_max_) is reached. Hence, the AF tests for the optimal adjustment of the neuromuscular system with regard to muscle tension and length in reaction to impacting loads. It was suggested that if muscle lengthening starts at a considerably low force level, joints might not be stabilized appropriately anymore. The strain on passive structures and on muscle origins and insertions consequently increases. This could result in a higher risk of injuries and complaints of the musculoskeletal system (Dech et al., 2021; Schaefer et al., 2021a; Schaefer et al., 2021b; Schaefer et al., 2022b). Therefore, the assessment of the maximal holding capacity is of major relevance in sports, movement and health sciences, and medicine.

Basic research on AF was executed using a technical measuring system based on pneumatics regarding its evaluation (quality criteria), providing first reference values (Schaefer et al., 2017; Dech et al., 2021) and concerning the effects of muscular pre-activation on the explosive AF (Schaefer and Bittmann, 2019). Dech et al. suggested differentiating the maximal holding capacity from commonly assessed maximal voluntary isometric contraction (MVIC) (Dech et al. (2021)). Both strength abilities were performed during isometric muscle action. However, during MVIC, the participant pushed against a resistance, whereas during maximal isometric holding actions (AFiso_max_), the participant reacted to the external load in a holding manner. AFiso_max_ was found to be significantly lower compared to MVIC in healthy participants when measured using a pneumatic device (Dech et al., 2021). Regarding the AF assessment using an objectified manual muscle test (MMT), healthy participants showed a significantly reduced AFiso_max_ in reaction to unpleasant imaginations (Schaefer et al., 2021b; Schaefer et al., 2022a) or odors (Schaefer et al., 2021a). The reduced holding capacity was interpreted as functional muscular instability. This instability was an instant effect in reaction to the negative stimuli and immediately switched back to stability by perceiving a positive stimulus (Schaefer et al., 2021a; Schaefer et al., 2021b; Schaefer et al., 2022a). Based on those findings, it was suggested that the holding capacity is particularly sensitive to disturbing or supporting inputs and might have potential for diagnostic approaches.

Little is known about the behavior of different torque parameters regarding repeated measurements. Ryan et al. investigated the effect of herbal supplements on the fatigability in five sets of 30 maximal isokinetic concentric contractions of leg extensors in three groups, including a placebo group (Ryan et al., 2021). The torque variables decreased in each group and plateaued at sets 4–5. Other researchers investigated the effect of repeated exercises on strength and other parameters over several days or weeks (Smith et al., 1994; Glowacki et al., 2004; Small et al., 2009; Gacesa et al., 2013), instead of effects on the same day as considered in the present study. Closely related to the AF or at least to HIMA is the so-called “eccentric quasi-isometric contraction” (EQI) (Oranchuk et al., 2019; 2021a; 2021b). This involves basically a submaximal HIMA with a constant force, which should be maintained for as long as possible in an isometric position until “fatigue causes muscle lengthening and then maximally resisting through a range of motion” (Oranchuk et al., 2019). During AF, in contrast, the external load increases further, whereby the participant has the task of maintaining the isometric position for as long as possible and merges into eccentric muscle action as soon as the maximal holding capacity is exceeded. To the knowledge of the authors, no repeated EQI measurements were investigated rather the effect of EQI resistance training. One of our own studies investigated the maximal AF measured using a pneumatic system over 50 repeated trials and found a stronger decline in five participants who indicated they performed power sports in contrast to five participants who stated they were active in endurance sports (Schaefer et al., 2017). This speaks for a stronger fatigability in power sport athletes. Other studies investigating different types of athletes focused mainly on force production or the mechanical properties of muscles (de Paula Simola et al., 2016). Mileva et al. investigated strength vs. endurance athletes regarding concentric peak force and MVIC after repeated sets (max. 15) of 15 repetitions of concentric knee extensions at 60% of their one-repetition maximum (Mileva et al., 2009). Strength athletes showed a stronger decline in force parameters than endurance athletes (Mileva et al., 2009).

Investigating the AF is of special relevance because it is more similar to motions in real life and sports than the usually assessed strengths. Hence, it is able to reflect the actual force capacity under those circumstances in a better way. Moreover, the assumed close connection between impaired holding capacity and injury mechanisms or the development of musculoskeletal complaints requires further research on AF.

The aim of this study was to investigate the behavior of different AF parameters (AFiso_max_ and AF_max_) over the course of 30 intermittently repeated trials. Since the AF has rarely been examined until now, little is known about the behavior regarding a high number of repetitions of such adaptive isometric–eccentric actions. The study should provide information on how many repetitions are possible without losing force. Insights and possible conclusions on physiological mechanisms should be obtained thereby, e.g., fatiguing effects. This has practical relevance for sports and for clinical practice, in which AF is assessed similarly by repeated MMTs. In addition, differences between maximal torque parameters, especially AFiso_max_ (maximal HIMA) vs. MVIC (maximal PIMA), should be examined. Moreover, first insights should be gained concerning possible differences between non-professional endurance and strength athletes regarding the torques generated over the course of 30 repetitions. The following research questions arose:

1) How do the AF parameters behave during repeated trials?
2) How does the MVIC react to repeated AF trials?
3) Does MVIC (maximal PIMA) differ from AFiso_max_ (maximal HIMA)?
4) Do the torque parameters differ in their behavior over the course of 30 repetitions when comparing endurance and strength athletes?

Different hypotheses have been raised: 1) AF_max_ and AFiso_max_ decrease significantly in the course of 30 trials, whereby AFiso_max_ shows a stronger decline than AF_max_. The latter was assumed because of the high sensitivity of AFiso_max_. 2) MVIC is significantly lower after compared to before AF trials. 3) AFiso_max_ is significantly lower than MVIC; thus, the previously suggested differentiation of two types of isometric muscle action will be verified. 4) All force parameters (AFiso_max_, AF_max_, and MVIC) are significantly lower in endurance athletes than strength athletes. This seems to be evident and is based on the knowledge that endurance athletes show lower torques than strength athletes (Clarkson et al., 1980; Häkkinen and Keskinen, 1989). 5) MVIC, AF_max_, and AFiso_max_ decrease stronger in strength vs. endurance athletes, since endurance athletes are assumed to be less fatigable.

The present study will provide novel insights regarding the behavior of AF with repetition and the MVIC after those repeated maximal adaptive isometric–eccentric activations. It should support and expand the basic understanding of the specific muscular holding function. Moreover, it should provide the first data on the behavior of the mentioned force parameters with respect to different types of sports. The findings will be relevant for sports, movement, training, health sciences, and physiological and neurological aspects of muscle function.

## 2 Materials and methods

The measurements took place at one appointment at the Neuromechanics Laboratory of the University of Potsdam (Germany). Elbow flexors were chosen for 30 intermittent repetition trials of AF, since this muscle group is often evaluated by the MMT in clinical practice. Furthermore, elbow flexors are especially trained in strength athletes, and hypertrophy effects can be expected. In contrast, endurance athletes are usually not exercising elbow flexors with respect to maximal strength. Hence, assessing elbow flexors seems to be suitable for providing first differences between those types of sports.

### 2.1 Participants

For the main consideration regarding the behavior of torque parameters (MVIC, AF_max_, and AFiso_max_) over the course of 30 trials, a repeated-measures design was present. To estimate the sample size *a priori* in G*Power (version 3.1.9.7, Düsseldorf, Germany), repeated measures ANOVA (RM ANOVA) within–between interaction with α = 0.05 and 1–β = 0.8 was chosen (correlation estimated as 0.9, non-sphericity correction: 1) the number of groups was 3 (torques), and the number of measurements was 2 (pre/post). To determine a substantial effect size of 0.8, a minimum of n = 6 participants was revealed. For interval consideration (30 trials were separated into six intervals), RM ANOVA within factors was chosen in G*Power with two groups (AF_max_ and AFiso_max_) and six measurements. A minimum sample size of n = 4 was revealed to detect a substantial effect size of 0.8. For slope comparison of AF parameters over the course of 30 trials, the dependent *t*-test (one-tailed, α = 0.05, 1–β = 0.8, and effect size = 0.8) was chosen in G*power and revealed a minimum sample size of n = 12. For comparative analyses between torque parameters, a paired *t*-test (one-tailed, α = 0.05 and 1–β = 0.8) was used. The calculation of effect size was based on the results from the study by Dech et al. regarding the mean and SD of differences in torques (AFiso_max_ vs. MVIC), resulting in d_z_ = 1.32 (Dech et al., 2021). A minimum of six participants was revealed to detect substantial differences between those parameters. Since the investigation of types of sports was only a side consideration, *a priori* analysis was not performed. With respect to those estimations, *n* = 12 participants were included in the present investigation.

In total, 12 healthy Caucasian males (age: 26.08 ± 3.42 yrs, height: 184.08 ± 4.08 cm, and body mass: 81.10 ± 9.17 kg) volunteered to participate in the study. Inclusion criteria were an involvement in sports at least three times per week, good overall health, and stable neuromuscular control of the assessed elbow flexors—examined by a preceding clinical muscle test in the sense of a break test (Conable and Rosner, 2011; Bittmann et al., 2020; Schaefer et al., 2022a) (explanations are given below). Exclusion criteria were complaints or disorders of the upper extremity, shoulder girdle, spine, or head within the last 6 months and any acute disease within the last 2 weeks (e.g., fever). All subjects were right-handed. The dominant arm was chosen for measurements in cases of stable neuromuscular control (*n* = 10 participants); otherwise, the non-dominant side was measured (*n* = 2).

According to the queried sports (see procedure), the sample was divided into two groups: strength athletes (*n* = 6; age: 24.83 ± 2.93 yrs; height: 185.67 ± 4.76 cm; body mass: 84.20 ± 6.68 kg; amount of resistance training: 274.00 ± 60.66 min/week, range: 200–360 min/week) and endurance athletes (*n* = 6; age: 27.33 ± 3.67 yrs; height: 182.50 ± 2.81 cm; body mass: 78.00 ± 10.83 kg; amount of endurance training: 370.00 ± 331.96 min/week, range: 180–1,040 min/week). Two participants from each group trained both endurance and strength. They were assigned to the groups according to the amount (min/week) of executed training. For both participants in the endurance group, the amount was four and seven times higher for endurance sports. For the two participants of the strength group, the amount was twice as high for resistance vs. endurance training.

The study was conducted according to the Declaration of Helsinki, and the permission of the local ethics committee of the University of Potsdam (Germany) was given (no. 39/2017; date: 30 November 2017). Each participant gave written informed consent.

### 2.2 Measurement system for recording the Adaptive Force

**FIGURE 1:**
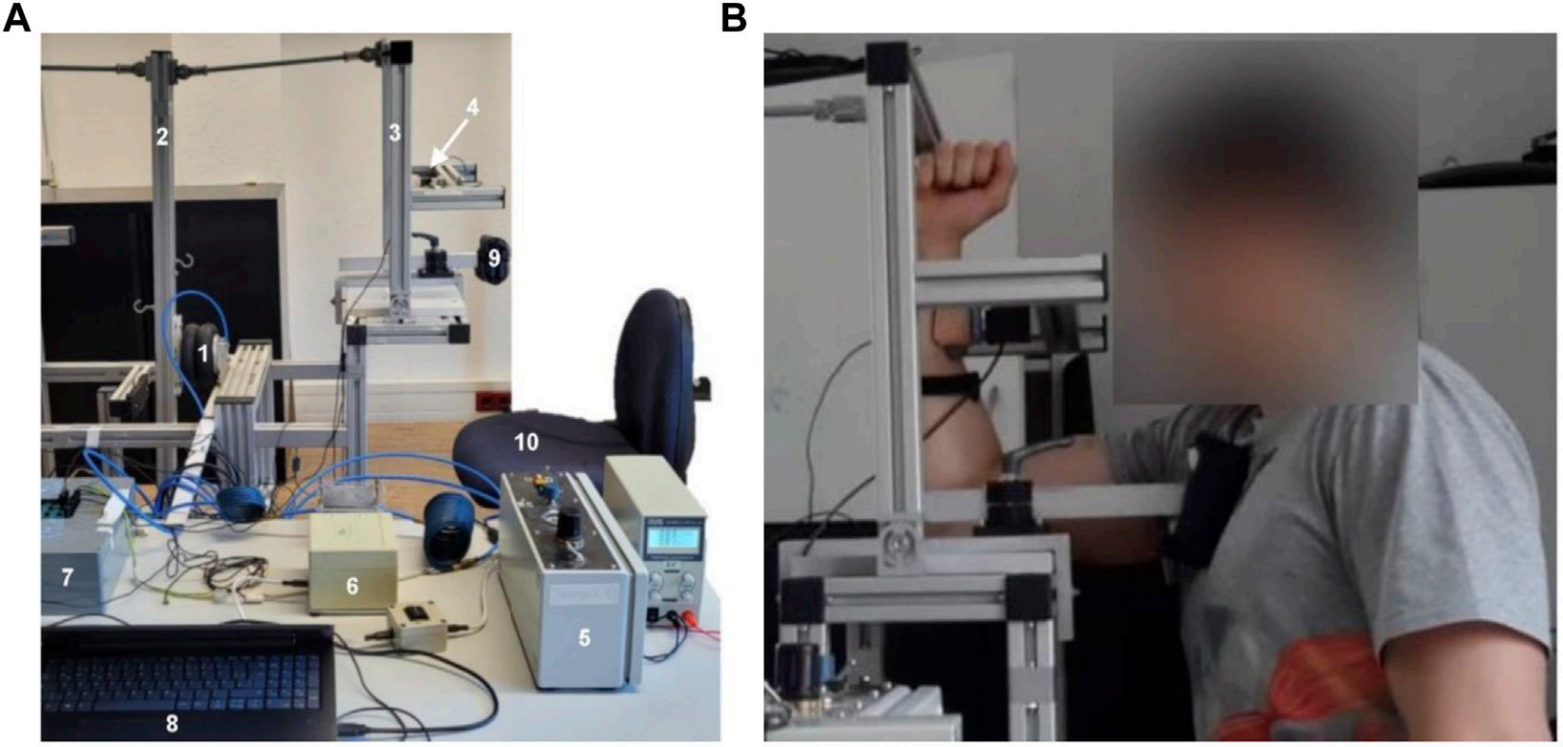
Measurement system and position. **(A)** Measurement system consists of a bellows cylinder (1), lever I (2), lever II (3), interface with a strain gauge (4), a control device including a throttle and pressure sensor (5), an additional motor throttle (6), an A/D converter (7), a measuring laptop (8), a cushion for thorax fixation (9), and a seating chair for the participant (10). The acceleration sensors and the compressor are not depicted. **(B)** Starting position to record the AF of the elbow flexors and the MVIC.

The pneumatically driven measurement system (Figure 1A) was used to detect the AF of elbow flexors and for MVIC tests. For the latter, the system was passive and fixed, but the strain gauge was the same. Specifications of the system components are given in Supplementary Table S1. The system consists of a compressor for the application of compressed air, a bellows cylinder, two levers (lever I in contact with the bellows cylinder and with lever II), an interface with the strain gauge at lever II, a control unit including a throttle and pressure sensor, an additional motor throttle to avoid an abrupt pressure increase at the beginning, two acceleration sensors (ACCs; one fixed at lever II and one at the participant’s forearm), an A/D converter, a measuring laptop including the software NI DIAdem 17.0 (National Instruments, Austin, TX, United States of America), a cushion for fixation of the thorax from anterior, and a chair for the participant. The measuring system was proven to be reliable and valid (unpublished). The evaluation of the partner system for measuring the AF of elbow extensors, which works with the identical chassis, bellows cylinder, compressor, and control device, revealed a high reliability (ICC = 0.896–0.966) with acceptable random errors (Dech et al., 2021).

The compressed air was led from the compressor to the bellows cylinder, which expanded. Due to the lever connection, lever II then moved away from the participant in the direction of elbow extension. Compressible air allowed the participant to maintain an isometric position until his maximal isometric AF was reached despite the impacting increasing load of the system. If the pressure exceeded the participant’s maximal holding capacity, he merged into eccentric muscle action. Thereby, he should still try to resist/decelerate the further increasing pressure as good as possible. A safety stop restricted a too-wide expansion of the bellows cylinder. The control unit regulated the velocity of pressure rise. The throttle to control the velocity of the pressure increase was adjusted individually for each participant (see below).

The strain gauge with the interface for contact with the participant’s forearm measured the force between the lever and the participant’s forearm. The two ACCs at lever II and the forearm recorded the angles. Force and ACC signals were transmitted through an A/D converter to the measuring laptop. The sampling rate was set at 1,000 Hz.

### 2.3 Setting

Figure 1B shows the position of the participant: he sat upright with both feet firmly on the ground, and the thorax fixation was placed on the measured side from the front beneath the clavicle to avoid a movement of the thorax during the measurement. The elbow joint was flexed 90° (controlled by a hydrogoniometer (MT.DOK; Desimed GmbH & Co. KG, Müllheim, Germany); range: 360° with 2° intervals), the shoulder was flexed ∼85° from the neutral zero position, and the forearm was in a vertical position. The rotation center of the participant’s elbow was placed in line with the pivot of the technical joint at the base of lever II. The interface was adjusted in height so that it contacted the forearm just beneath the radial styloid process. The center of the interface was used to determine the lever length. The second ACC was fixed beneath the ulnar styloid process with double-sided tape to measure the motion of the forearm.

### 2.4 Procedure

At the beginning of each measurement day, a calibration of the measuring system took place to standardize the system pressure. Every subject performed the measurement series during a single appointment. It was guided by two testers: the first one controlled the device/software (NI™ DIAdem (National Instruments, Austin, TX, United States), and the second one was responsible for placing the participant and readjusting the lever after each trial*.* All participants were informed by a written participant information brochure and by a verbal introduction to the measurement procedure and system. Afterward, they gave their written informed consent and answered a questionnaire regarding biometric data, sports activities (type, amount/week), previous injuries of the upper extremities, current condition and complaints, handedness, and physical activity within the last 24 h.

Subsequently, the neuromuscular control of the elbow flexors of each participant was checked individually by two experienced examiners using the clinical manual muscle test (Bittmann et al., 2020; Schaefer et al., 2021a; Schaefer et al., 2021b; Schaefer et al., 2022a; Schaefer and Bittmann, 2022). In case the participant was able to maintain the isometric position during the entire external force increase applied by the examiners manually, the neuromuscular control was rated as stable as one inclusion criterion (see above). If the muscle lengthened in the course of the force increase, the test was assessed as unstable. In two cases, the elbow flexors on the dominant side did not show proper stability; hence, the non-dominant side, which was rated as stable, was regarded for AF measurements.

Afterward, the device was adjusted according to the individual measurement position (i.e., height of chair, lever length, and thorax fixation). An overall warm-up was executed as follows: mobilization of upper extremities and low intensity muscle warm-up (curls) with two sets (resting period: 1 min) of 20 repetitions with dumbbells (5 or 6.25 kg). Afterward, the participant was positioned in the measuring system, and the ACC sensor was fixed to the forearm. The starting (∼90°) and end positions (contact at the security stop; total range from start to end: ∼20°) of the forearm and lever length were recorded for reference. A specific warm-up of the elbow flexors followed. For that, the pressure system was closed in the most extended, thus, end position of the bellows cylinder, so air was in the system but could not stream out. The participant performed concentric contractions of the elbow flexors by pushing the forearm against the interface up to a self-estimated half of the maximal force (2 × 10 repetitions, 1-min resting period). The resistance given by the air in the bellows cylinder increased thereby, since the bellows cylinder was compressed by the muscular activity of the participant. The system was completely passive in this process.

The measurement series started with three MVIC trials (MVICpre), where the lever was fixed and the participant had to push with maximal force against the interface. The system was passive; it just provided a stable abutment. The participant should increase the force smoothly, reach his maximum within 3 s, and maintain this for 1–2 s. An abrupt and powerful force increase was avoided. The resting period was 60 s. MVIC was used to adjust the throttle individually. This was set so that 70% of the MVIC was reached after 3 s under stable conditions. Subsequently, 30 AF measurements were performed consecutively using that throttle configuration. During the trials, the pneumatically driven lever pushed against the participant’s forearm in the direction of elbow extension. The participant’s task was to maintain the starting position in an isometric holding manner for as long as possible despite the increase in pressure in the system. Thus, he should adapt isometrically to the impacting increasing load. The pressure rose to an amount that overwhelmed each participant. Therefore, each participant was forced into eccentric muscle action of the elbow flexors as soon as the external pressure exceeded his maximal holding capacity (AFiso_max_). In this eccentric phase, the participant still had the task of resisting/decelerating the increasing load as good as possible. A trial was finished as soon as the lever reached the end position or if the participant stopped the resistance. The resting period between the trials was 120 s. After the 30 AF trials, two MVIC tests (MVICpost) were performed again to compare the MVIC before and after the AF trials. The whole appointment lasted for ∼2 h. The subjective general exhaustion was queried on a scale from 0 to 10 after each AF trial.

### 2.5 Data processing and statistical analyses

Data recording and processing were carried out using the software NI™ DIAdem (National Instruments, Austin, TX, United States). To prepare the data for evaluation, ACC signals were converted from volts to angles. All raw signals (force, pressure, ACC) were filtered with low pass Butterworth filter (filter order: 10, cutoff frequency for force and pressure: 3 Hz, for ACC: 1 Hz). Thereupon, the following force parameters were extracted. It is to be noted that the force was recorded in V and was transformed after extraction into torque (Nm) by using the formula M = F *r, where F is the force in N (converted by 1 V = 19.886 kg * 9.81 = 195.082 N) and r is the length of the individual rotational axis (lever) in m.

1) Maximal voluntary isometric contraction (MVIC)

The peak values of the MVIC trials were extracted. The highest values of the three MVIC trials before and of the two MVIC trials after the AF measurements were chosen as maxMVICpre (Nm) and maxMVICpost (Nm), respectively, and were used for further consideration.

2) Parameters of Adaptive Force

**FIGURE 2:**
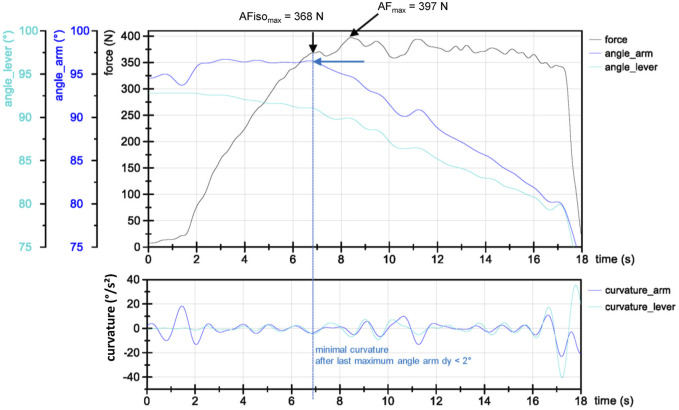
Exemplary force (N) and angle signals (in degree, converted from ACC of the forearm (arm, blue) and lever (turquoise) (bottom) and the second derivation of angle signals (curvature; below)) of one AF measurement (second trial) of a strength athlete. AFiso_max_ and AF_max_ are highlighted. AFiso_max_ is the force value at the minimal curvature after the last maximum in angle signal (here, forearm) with dy < 2°, where the lever angle does not show a pushing action of the participant. After AFiso_max_, both angle signals decrease with some phases of minor concentric or short isometric actions.

Exemplary force and angle signals of the arm and lever for one AF measurement are shown in Figure 2, illustrating the main aspects of the evaluation of AF parameters.

2.1) Maximal Adaptive Force (AF_max_)

The peak value of each AF trial is referred to as AF_max_ (Nm).

2.2) Maximal isometric Adaptive Force (AFiso_max_)

AFiso_max_ (Nm) defines the force value at the moment of first yielding of the forearm (breaking point). To determine this, a standardized algorithm was used according to Dech et al. (2021). The main criterion for AFiso_max_ was that a holding isometric action was present from the beginning of the measurement. Thus, the necessary defined conditions were yielding of the forearm ≤2° (isometric action is still acceptable) and a push back of lever I ≤ 0.3° (pushing isometric action or concentric muscular action were excluded thereby, which was not the case in the present study). The limit values have been set in previous investigations (Dech et al., 2021). To determine the exact breaking point, the angles of the arm and lever were used. The second derivation was calculated from these to find the point of greatest curvature. AFiso_max_ was defined as the highest force value between the last maximum in angle signals (arm or lever) and the point of the subsequent greatest curvature before the forearm yielded more than 2° (arm angle). The deviation of the forearm (arm angle) at the beginning as shown in Figure 2 occurred regularly as soon as the force increased. It was presumably due to the cushion of the interface, the participant’s hand, elbow, or shoulder joint. Lever I did not show this behavior; on the contrary, its angle showed a yielding mostly always from the beginning. However, it was decisive that the forearm was in an isometric position. Due to the still-novel algorithm, the determined AFiso_max_ values were also checked visually. In 359 of 360 trials, the detected AFiso_max_ corresponds to the visual assessment of the breaking point.

Different ratios were calculated for further consideration to gather information on the relation of torque parameters: $\frac{{AFiso}_{\max}}{{AF}_{\max}}$ (%), $\frac{{AFiso}_{\max}}{maxMVICpre}$ (%), and $\frac{{AF}_{\max}}{maxMVICpre}$ (%).

### 2.6 Statistical analyses

The statistical analyses were executed using SPSS Statistics 29 (IBM, Armonk, New York, United States) and Excel (Office 365, Microsoft, Redmond, Washington, United States). Different approaches were used according to the research questions mentioned in the introduction. Basically, all data were normally distributed as confirmed by the Shapiro–Wilk test. Parametric tests were chosen for statistical comparisons (see below). In this case, RM ANOVA was used, and sphericity was checked by Mauchly’s test. In case of significance, the Greenhouse–Geisser correction was applied (F_G_). Cohen’s effect size f was given for RM ANOVA, where Cohen’s f was calculated by $\sqrt{\frac{\eta^{2}}{1-\eta^{2}}}$ (Cohen, 1988). For t tests (paired or unpaired), Hedges’ effect size g was calculated by SPSS. The effect sizes were interpreted as small (0.2), moderate (0.5), large (0.80), or very large (1.3) (Cohen, 1992; Sullivan and Feinn, 2012). Significance level was α = 0.05.

1) Behavior of force parameters with respect to repeated AF measurements (*n* = 12):

The linear mixed model (method: restricted maximum likelihood; REML) was used to investigate the maximal torques regarding time (pre/post or start/end, respectively) and parameter (AF_max_, AFiso_max_, and MVIC). ‘Time’ and ‘parameters’ were set as fixed factors. Since time*parameter was not found to be significant in terms of fixed factors, it was removed from the mixed model in order to reduce complexity. ‘Subject’ (ID) and ‘parameter’ were defined as random effects for the first estimation. Since ‘parameter’ turned out to be not significant in covariance estimation, consideration of the random factor was not necessary (Baltes-Götz, 2020). The Kenward–Roger approximation was used to estimate the degrees of freedom (df) since it provides a better estimation for small sample sizes (Baltes-Götz, 2020). This model revealed the best Bayesian information criterion (BIC) and was therefore used.

The ratio of AFiso_max_ to AF_max_ was compared between start and end by the paired *t*-test. Relative declines of parameters from pre to post or start to end (%), respectively, were calculated and compared using the paired *t*-test for each parameter (one-tailed test for AFiso_max_ vs. AF_max_ or MVIC, since AFiso_max_ was assumed to decrease stronger; two-tailed test for AF_max_ vs. MVIC).

Slopes of regression lines of the single values of each parameter (AF_max_ and AFiso_max_) regarding the 30 AF measurements (M1–M30) were calculated to describe a possible decline during repetition trials. The paired *t*-test (one-tailed) was performed to investigate a possible difference between the slopes of AFiso_max_ and AF_max_.

Furthermore, the 30 AF trials were divided into six intervals (I1–I6), which consisted of the arithmetic mean of each five subsequent trials: I1 (M1–M5), I2 (M6–M10), I3 (M11–M15), I4 (M16–M20), I5 (M21–M25), and I6 (M26–M30). RM ANOVAs for every AF parameter considering the six intervals were performed. Pairwise comparisons were executed using the Bonferroni correction (adjusted *p*-value = p_adj_).

2) Comparison of force parameters (*n* = 12)

The comparison of maxAFiso_max_ and maxMVICpre is most important regarding the differentiation of HIMA and PIMA. maxAFiso_max_ and maxAF_max_ refer to the highest value of all 30 trials regarding AFiso_max_ and AF_max_, respectively. The paired *t*-test was used to check for differences between the maximal torques.

3) Comparisons between sports groups (endurance vs. strength athletes)

This consideration has to be regarded as preliminary due to the small sample sizes of both groups. Differences regarding the overall maximal torques of MVIC, AF_max_, and AFiso_max_ between endurance and strength athletes were checked by unpaired *t*-tests (one-tailed) for each parameter separately.

The relative declines (%) of maxMVIC from pre to post and of AF_max_ and AFiso_max_ from start to end were calculated and compared between both sports groups using unpaired t-tests (one-tailed).

The slope values of the linear regression line of AF parameters were used to test for differences between endurance and strength athletes by performing unpaired t tests (one-tailed). The comparisons of the slope of regression lines regarding the ratios were considered as well. This should provide information on the assumed different behaviors of athletes with respect to torque relations.

For the six intervals, a REML was executed for AF_max_ and AFiso_max_. Both parameters were considered separately since only the effect of sports types was of interest. ‘Interval’ was regarded as a covariate. Fixed factors were ‘sports’ (endurance and strength), ‘intervals’ (I1–I6), and sports*interval. ‘ID’ and ‘interval’ were set as random factors (unstructured).

Regarding the patterns of decline, the averaged torques of I1 (AF_max_ and AFiso_max_, respectively) were set at 100%, and the values of the subsequent intervals were related to the first value. Differences between strength and endurance athletes were checked by a mixed ANOVA (intervals*sports). In the case of significance, pairwise comparisons were performed.

## 3 Results

The single values of each participant and trial for MVIC, AF_max_, and AFiso_max_ including arithmetic means, standard deviations, and coefficients of variation are given in Supplementary Tables S1–S4.

### 3.1 Behavior of torque parameters with respect to repeated AF measurements

#### 3.1.1 Maximal torques with respect to repeated measurements

**FIGURE 3:**
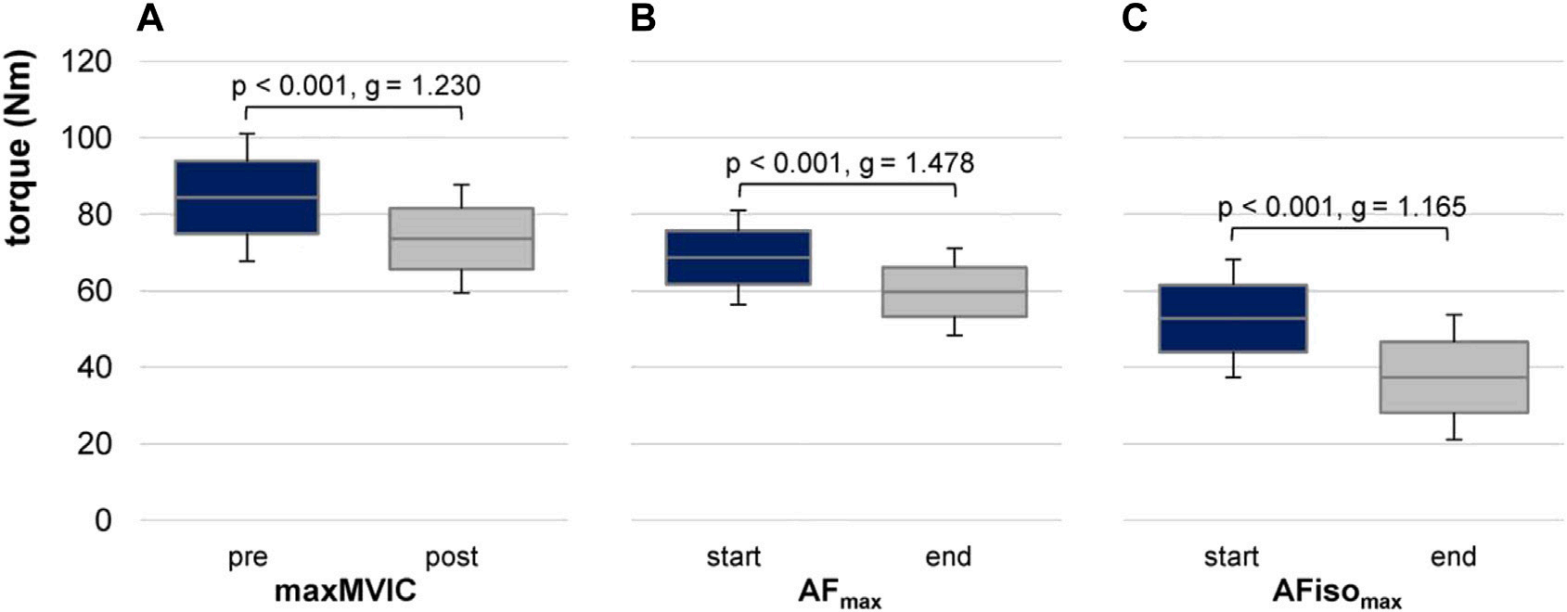
Maximal torques at pre/post and start/end of 30 AF trials. Arithmetic means, standard deviations (error bars), and 95% confidence intervals (CIs) are depicted for maximal values of **(A)** MVIC before (pre) and after (post) AF trials (maxMVIC), **(B)** maximal Adaptive Force (AF_max_), and **(C)** maximal isometric AF (AFiso_max_) at start and end. *p*-values and effect sizes of Hedges’ g are given for pairwise comparisons.

Considering all *n* = 12 participants, maxMVICpre amounted to 84.39 ± 16.68 Nm and maxMVICpost to 73.62 ± 14.10 Nm (Figure 3). The duration to reach MVIC was, on average, 3.36 ± 1.00 s.

The maximal value of AF_max_ at start (within the first three trials) amounted to 68.72 ± 12.35 Nm and 59.73 ± 11.38 Nm at end (within the last three trials). The maximal value of AFiso_max_ at start was 52.78 ± 15.45 Nm and 37.38 ± 16.35 Nm at end (Figure 3). The average duration to reach AF_max_ was 11.92 ± 2.12 s and 3.98 ± 0.90 s for AFiso_max_.

The linear mixed model estimated an ICC of ρ = 0.677. MVIC was significantly higher than AFiso_max_ (t = 14.253, *p* < 0.001, 95% CI for the difference was 29.16–38.69), analog for AF_max_ vs. AFiso_max_ (t = 8.042, *p* < 0.001, 95% CI = 14.375–23.908). The torques were significantly higher for pre vs. post (t = 6.030, *p* < 0.001, 95% CI = 7.83–15.61). The random subject variance was 142.8 (SD 11.95). Pairwise comparisons revealed significant differences between pre and post for all torque parameters (MVIC: *p* < 0.001 and g = 1.165; AF_max_: *p* < 0.001 and g = 1.478; AFiso_max_: *p* < 0.001 and g = 1.165). Parameter*time turned out to be non-significant (*p* = 0.186 to *p* = 0.336).

$\frac{{AFiso}_{\max}}{{AF}_{\max}}$ amounted to 77.39% ± 11.36% at start and 64.10% ± 19.21% at end of 30 trials, which differed significantly (t (11) = 2.527, *p* = 0.014, and g = 0.678).

Comparing the relative declines of MVIC pre/post, AF_max_, and AFiso_max_ start/end, they were similar for MVIC and AF_max_ with −12% and −13%, respectively, but clearly stronger for AFiso_max_ with −29%. Pairwise t tests revealed significant differences between the relative declines of AFiso_max_ vs. AF_max_ (*p* = 0.014 and g = 0.675) and of AFiso_max_ vs. MVIC (*p* = 0.041 and g = 0.513); AF_max_ and MVIC were not significantly different (*p* = 0.877).

The increase of general exhaustion on the scale from 0 to 10 from the first (1.62 ± 0.96, *n* = 12) to the last AF trial (3.46 ± 2.03, *n* = 12) was 1.85 ± 2.08, whereby one strength athlete showed a clear rise from start (3) to end (9).

#### 3.1.2 AF parameters in the course of 30 repetitions

**FIGURE 4:**
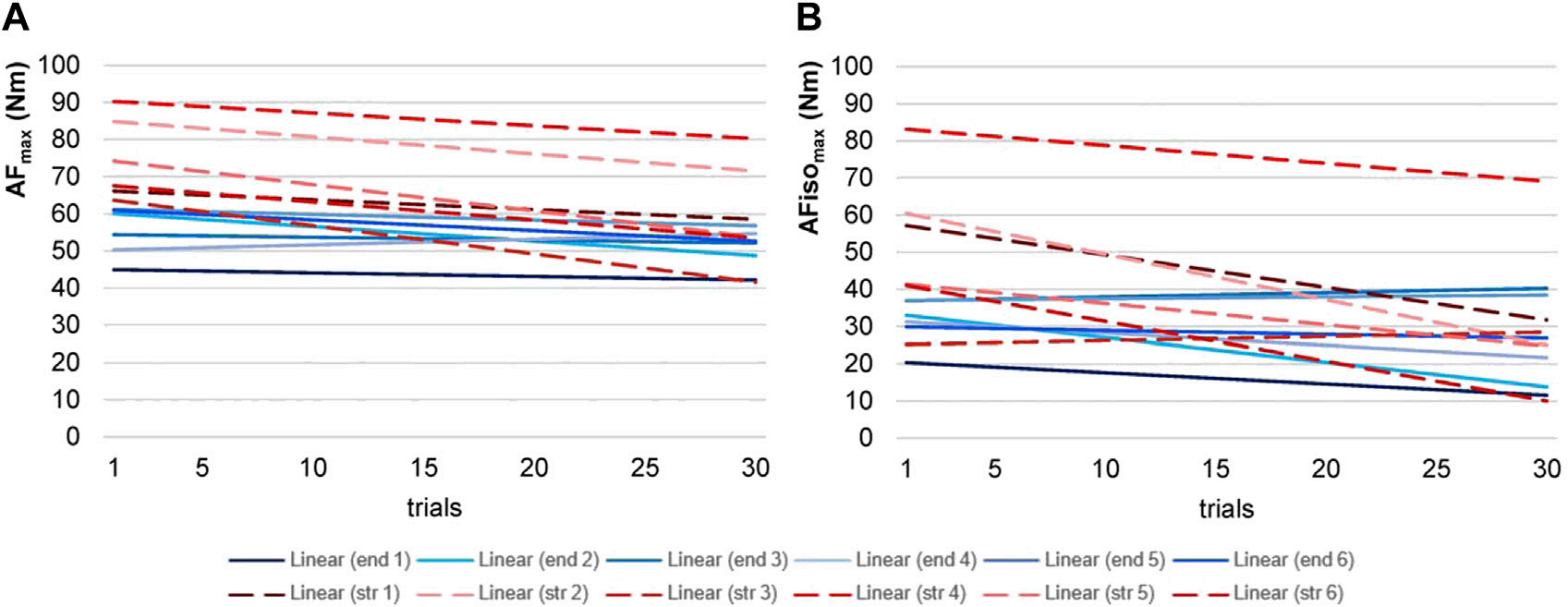
Regression lines of Adaptive Force parameters. Displayed are the regression lines of the torques of maximal Adaptive Force (AF_max_, **(A)**) and maximal isometric AF (AFiso_max_, **(B)**) of the 30 trials of each participant (endurance = end (blue), *n* = 6; strength = str (red, dashed), *n* = 6).

The regression lines of the 30 AF_max_ and AFiso_max_ values of each participant are illustrated in Figure 4. Averaged over the *n* = 12 participants, the slopes were 0.32 ± 0.26 for AF_max_ and 0.45 ± 0.45 for AFiso_max_, which did not differ significantly (t (11) = 0.910, *p* = 0.191).

**FIGURE 5:**
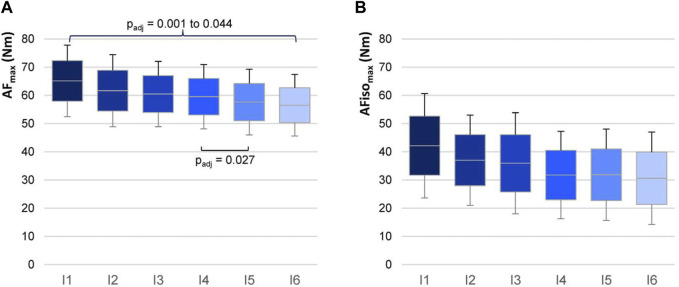
AF torques in six intervals. Arithmetic means, standard deviations (error bars), and 95% CIs of AF_max_ **(A)** and AFiso_max_ **(B)** regarding the six intervals (I1–I6) are illustrated for n = 12 participants. Intervals consist of the arithmetic mean of torques for five consecutive measurements each (I1 = mean of M1 to M5; … I6 = mean of M25 to M30). Adjusted *p*-values (Bonferroni correction) are given for significant pairwise comparisons.

Table 1 and Figure 5 illustrate the results regarding the intervals. AF_max_ decreased rather continuously with averagely −2.8% ± 1.6% per interval (range: −5.4% to −1.6%). AFiso_max_ showed a stepwise decline from I1 to I2 (−12.1%) and from I3 to I4 (–11.6%); from I2 to I3 and from I4 to I6, the decrease was considerably lower with an average of −2.2% ± 2.2% (range: −3.9% to +0.3%). RM ANOVA comparing the six intervals revealed a significant main effect for AF_max_ (F_G_ (1.57, 17.27) = 12.276, *p* = 0.001, f = 1.056). Pairwise comparisons with the Bonferroni correction showed that I1 differed significantly from all other intervals (I1 vs. I2: p_adj_ = 0.044, g = 1.022; I1 vs. I3: p_adj_ = 0.004, g = 1.435; I1 vs. I4: p_adj_ = 0.001, g = 1.657; I1 vs. I5: p_adj_ = 0.001, g = 1.735; I1 vs. I6: p_adj_ = 0.017, g = 1.174). I2 and I3 showed no significant differences regarding the subsequent intervals with the applied Bonferroni correction. I4 and I5 differed significantly (p_adj_ = 0.027, g = 1.095). For AFiso_max_, a significant main effect was found as well (F_G_ (2.41, 26.54) = 4.487, *p* = 0.016, f = 0.639). However, the Bonferroni-corrected pairwise comparisons showed no significant differences (p_adj_ = 0.069–1.000), presumably due to the high variation.

**TABLE 1:** Torque values regarding intervals and percentage decline. Arithmetic means (M) and standard deviations (SD) of maximal voluntary isometric contraction (MVIC, Nm) before (pre) and after (post) AF trials, maximal isometric Adaptive Force (AFiso_max_, Nm), maximal AF (AF_max_, Nm), and their ratios regarding the six intervals (I1–I6) considered for the whole sample (*n* = 12) and for each sports group separately (endurance, strength) are displayed. Furthermore, M ± SD of the percentage decline (%) from pre to post for maxMVIC and from start (max. of the first three trials) to end (max. of the last three trials) for AF parameters is given.

**Parameter**	**Time / interval**	**M ± SD**		
		**Total (*n* = 12)**	**Endurance (*n* = 6)**	**Strength (*n* = 6)**
maxMVIC (Nm)	pre	84.392 ± 16.683	76.045 ± 10.296	92.739 ± 18.414
	post	73.620 ± 14.101	63.938 ± 9.394	83.303 ± 11.144
	% decline	−12.347 ± 8.911	−15.750 ± 8.150	−8.945 ± 8.971
AFiso_max_ (Nm)	I1	42.161 ± 18.504	31.973 ± 6.733	52.349 ± 21.421
	I2	37.049 ± 16.001	29.034 ± 9.211	45.063 ± 18.007
	I3	35.938 ± 17.930	28.152 ± 11.399	43.725 ± 20.780
	I4	31.770 ± 15.510	27.304 ± 8.847	36.237 ± 20.077
	I5	31.871 ± 16.199	27.685 ± 10.171	36.057 ± 20.780
	I6	30.621 ± 16.427	26.165 ± 12.081	35.077 ± 20.001
	% decline	−28.734 ± 24.716	−25.239 ± 30.452	−32.228 ± 19.679
AFmax (Nm)	I1	65.161 ± 12.687	56.700 ± 6.039	73.630 ± 12.069
	I2	61.677 ± 12.798	52.640 ± 6.825	70.720 ± 10.848
	I3	60.488 ± 11.567	53.090 ± 4.995	67.890 ± 11.744
	I4	59.551 ± 11.412	52.700 ± 5.578	66.400 ± 11.952
	I5	57.630 ± 11.640	51.490 ± 5.434	63.770 ± 13.348
	I6	56.519 ± 10.925	52.680 ± 5.017	60.350 ± 14.216
	% decline	−12.840 ± 8.321	−10.488 ± 10.095	−15.191 ± 6.094
AFiso_max_ / AF_max_	I1	0.626 ± 0.169	0.557 ± 0.096	0.694 ± 0.205
	I2	0.582 ± 0.148	0.539 ± 0.127	0.625 ± 0.166
	I3	0.572 ± 0.195	0.521 ± 0.193	0.623 ± 0.201
	I4	0.522 ± 0.177	0.513 ± 0.143	0.532 ± 0.220
	I5	0.547 ± 0.210	0.530 ± 0.168	0.563 ± 0.261
	I6	0.521 ± 0.187	0.489 ± 0.195	0.554 ± 0.191
AFiso_max_ / maxMVICpre	I1	0.497 ± 0.163	0.424 ± 0.086	0.570 ± 0.196
	I2	0.436 ± 0.141	0.387 ± 0.135	0.485 ± 0.141
	I3	0.423 ± 0.176	0.381 ± 0.190	0.465 ± 0.166
	I4	0.375 ± 0.150	0.366 ± 0.145	0.383 ± 0.168
	I5	0.377 ± 0.162	0.369 ± 0.150	0.385 ± 0.189
	I6	0.354 ± 0.142	0.345 ± 0.155	0.363 ± 0.143
AF_max_ / maxMVICpre	I1	0.781 ± 0.115	0.754 ± 0.113	0.807 ± 0.121
	I2	0.740 ± 0.132	0.702 ± 0.128	0.779 ± 0.135
	I3	0.727 ± 0.116	0.707 ± 0.107	0.746 ± 0.131
	I4	0.716 ± 0.114	0.702 ± 0.108	0.729 ± 0.129
	I5	0.693 ± 0.120	0.686 ± 0.106	0.701 ± 0.142
	I6	0.681 ± 0.113	0.699 ± 0.082	0.662 ± 0.143

### 3.2 Comparison of maximal torques

Although both, MVIC and AFiso_max_, were executed during isometric actions, maxAFiso_max_ amounted to 56.86 ± 16.21 Nm and was significantly lower than maxMVICpre with 84.39 ± 16.68 Nm (t (11) = 7.105, *p* < 0.001, g = 1.907). Hence, maximal HIMA (AFiso_max_) was only 67.67% ± 13.60% of maximal PIMA (MVIC). Furthermore, a significantly lower maxAFiso_max_ than maxAF_max_ = 70.36 ± 12.27 Nm appeared with a relation of 80.07% ± 12.54% (t (11) = 5.593, *p* < 0.001, g = 1.502). Moreover, maxAF_max_ amounted to 84.38% ± 10.84% of maxMVICpre, which was also significantly lower (t (11) = 4.777, *p* < 0.001, g = 1.282). Most important here is that maximal HIMA was found to be significantly lower than maximal PIMA, although both muscle actions were isometric with the same muscle length.

### 3.3 Comparison between sports groups

#### 3.3.1 Maximal torques comparing endurance vs. strength athletes

MaxMVICpre amounted to 76.04 ± 10.30 Nm for endurance athletes and 92.74 ± 18.41 Nm for strength athletes; maxMVICpost was 63.94 ± 9.39 Nm for endurance athletes and 83.30 ± 11.14 Nm for strength athletes. Both parameters were significantly lower in endurance than strength athletes (maxMVICpre: t (10) = −1.938, *p* = 0.041, g = 1.033; maxMVICpost: t (10) = −3.254, *p* = 0.004, g = 1.734). The maximal torque of AF_max_ at start was significantly lower for endurance athletes compared with strength athletes (61.94 ± 5.61 Nm vs. 75.50 ± 13.93 Nm; t (6.58) = −2.211, *p* = 0.033, g = 1.178). At the end of the 30 AF trials, maxAF_max_ amounted to 55.03 ± 3.09 Nm for endurance athletes and 64.43 ± 14.90 Nm for strength athletes, which did not differ significantly (*p* = 0.093). The torques of AFiso_max_ at start amounted to 46.32 ± 11.30 Nm vs. 59.23 ± 17.24 Nm for endurance athletes vs. strength athletes and 33.96 ± 12.81 Nm vs. 40.81 ± 19.90 Nm at end, respectively. However, AFiso_max_ did not differ significantly between endurance and strength athletes, presumably due to the high variance. In summary, endurance athletes showed −15% to −23% lower torques than strength athletes.

#### 3.3.2 Decline of torques comparing endurance vs. strength athletes

Strength vs. endurance athletes showed a lower decline in MVIC from pre to post (−8.94% ± 8.97% vs. −15.75% ± 8.15%). In contrast, strength vs. endurance athletes showed a stronger decline for AF_max_ and AFiso_max_ from start to end, respectively (AF_max_: −15.19% ± 6.09% vs. −10.49% ± 10.10%; AFiso_max_: −32.23% ± 19.68% vs. −25.24% ± 30.45%). With the given sample sizes, the differences turned out to be non-significant (*p* = 0.100–0.323).

Regarding the slopes of the regression lines over the course of 30 AF trials (Figure 4), five endurance athletes showed a slight decrease, and one athlete showed an increase for AF_max_ (−0.14 ± 0.19; range: −0.29 to +0.15). All six strength athletes showed a decline (−0.50 ± 0.20; range: −0.76 to −0.26). For AFiso_max_, an increase for two endurance athletes was found, while the other four decreased (−0.21 ± 0.29; range: −0.66 to +0.12). One strength athlete showed an increase, while the other five showed a decrease (−0.68 ± 0.48; range: −1.22 to +0.11). The declines were significantly steeper for strength vs. endurance athletes regarding all parameters (AF_max_: t (10) = 3.264, *p* = 0.004, g = 1.739; AFiso_max_: t (10) = 2.098, *p* = 0.031, g = 1.118; $\frac{{AFiso}_{\max}}{{AF}_{\max}}$ : t (10) = 3.707, *p* = 0.002, g = 1.975; $\frac{{AF}_{\max}}{maxMVICpre}$ : t (10) = 2.597, *p* = 0.013, g = 1.384), except for $\frac{{AFiso}_{\max}}{maxMVICpre}$ , which slightly missed significance (*p* = 0.059).

**FIGURE 6:**
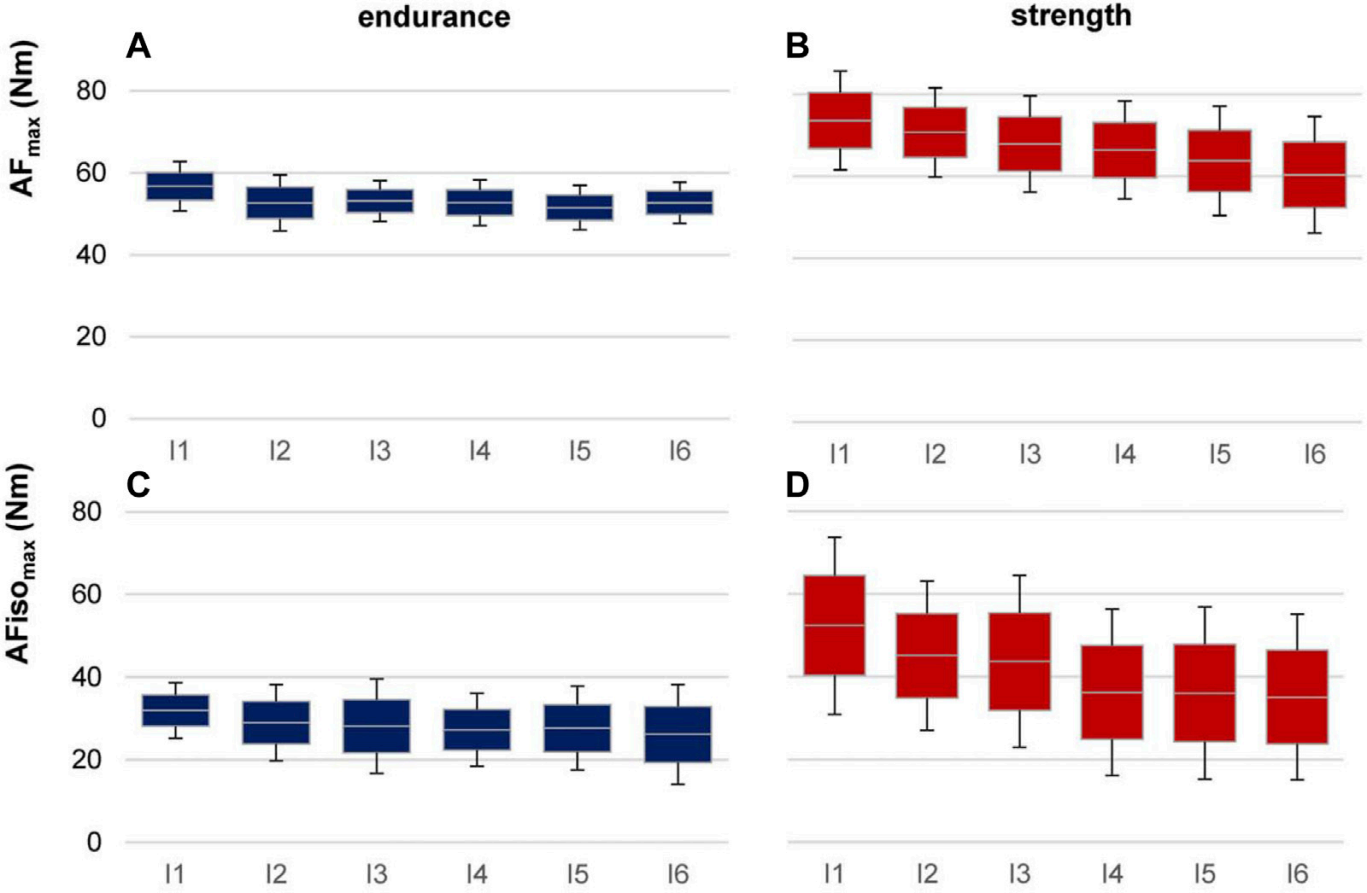
AF torques in six intervals for strength vs. endurance athletes. Displayed are the arithmetic means, standard deviations (error bars), and 95% CIs of AF_max_ **(A, B)** and AFiso_max_ **(C, D)** for endurance (left, blue, *n* = 6) and strength (right, red, *n* = 6) athletes regarding the six intervals of 30 trials (I1–I6).

Regarding the six intervals, it was evident that the patterns that were found considering the whole group were sharpened by considering the sports groups (Figure 6); strength athletes showed a rather constant decline in AF_max_, whereas endurance athletes more or less stayed on a similar level. The linear mixed model revealed an ICC of ρ = 0.957. Sports, intervals, and sports*intervals were significant in fixed-factor estimation. Endurance athletes showed a significantly lower torque than strength athletes (t = −3.927, *p* = 0.003, 95% CI for the difference was from −31.960 to −8.821). Regarding sports*interval, averaged torque decrease was estimated with −2.534 for strength athletes (t = −6.447, *p* < 0.001, 95% CI = −3.410 to −1.568). Endurance athletes decreased significantly less than strength athletes with an estimated value of −0.682 (t = 3.332, *p* = 0.008, 95% CI = 0.613–3.090). The variance of torque revealed a significant result in estimates of covariance parameters (Wald Z = 2.139, *p* = 0.032). The variance of slope and covariance were not significant. Pairwise comparisons showed that each interval, except for the last one (I6), differed significantly between endurance and strength athletes (*p* = 0.003–0.032, g = 1.111–1.841). For AFiso_max_, endurance athletes showed a similar pattern as in AF_max_ by staying on a similar level regarding the six intervals. In contrast, strength athletes developed a plateau formation in the sense that after I1 and I3, AFiso_max_ decreased erratically. The linear mixed model revealed an ICC of ρ = 0.836. Endurance athletes showed significantly lower torque than strength athletes (t = 2.444, *p* = 0.035, 95% CI = −41.530 to −1.921). Sports*intervals revealed an estimated averaged torque decrease of −3.453 for strength athletes (t = −4.431, *p* < 0.001, 95% CI = −5.190 to −1.717). Endurance athletes decreased significantly less than strength athletes with an estimated value of −0.970 (t = 2.484, *p* = 0.048, 95% CI = 0.028–4.939). Estimates of covariance parameters were non-significant (*p* = 0.058–0.868). Pairwise comparisons revealed that AFiso_max_ differed significantly between both groups only for the first two intervals (I1: *p* = 0.025, g = 1.184; I2: *p* = 0.040, g = 1.034). I3 to I6 showed no significant differences between strength and endurance athletes.

**FIGURE 7:**
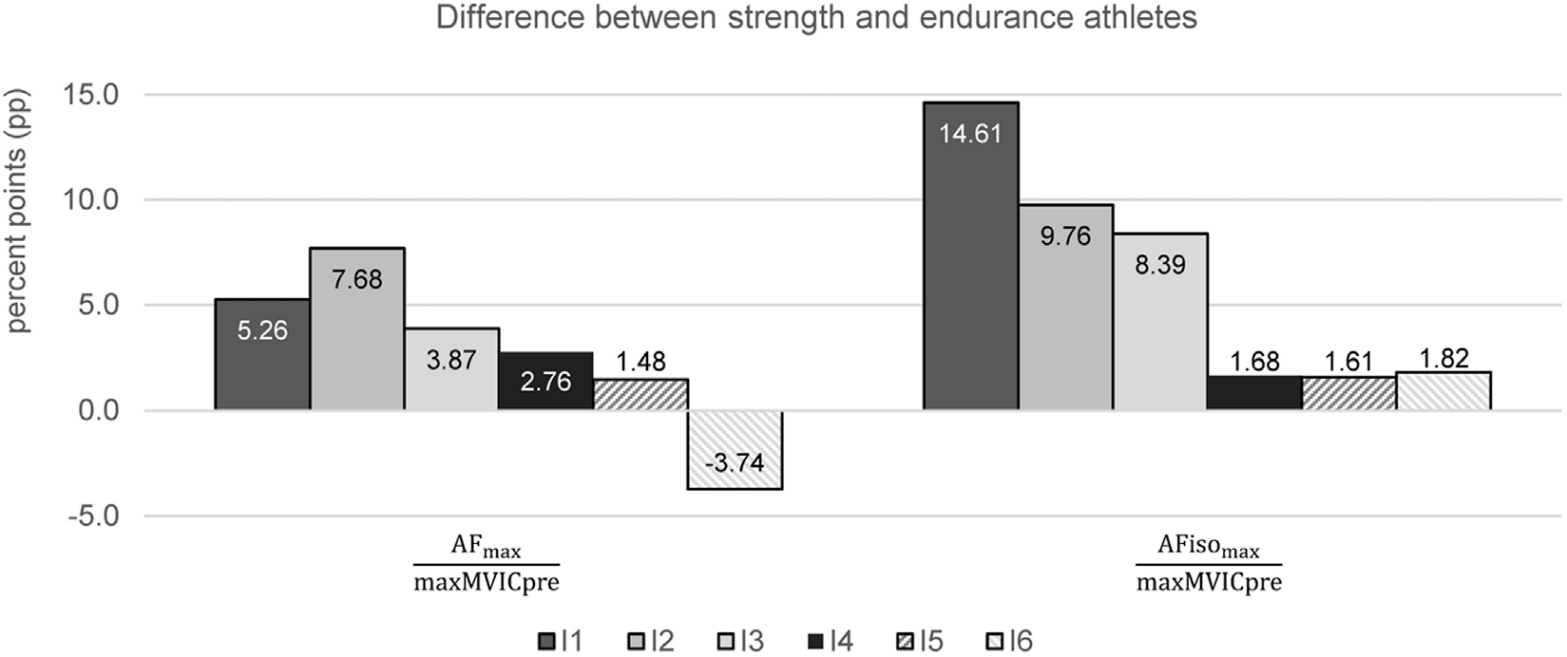
Differences between strength and endurance athletes for the ratios of AF_max_ to maxMVICpre and AFiso_max_ to maxMVICpre regarding each interval (I1–I6) in percent points (pp). Positive values indicate higher ratios for strength athletes. No difference turned out to be significant.

It seems worth mentioning that strength athletes showed a higher ratio of $\frac{{AF}_{\max}}{maxMVICpre}$ than endurance athletes in all intervals except for the last one (Figure 7). I6 was lower in strength athletes than in endurance athletes by −3.74 percent points (pp). For $\frac{{AFiso}_{\max}}{maxMVICpre}$ , the differences converged over the course of repeated trials. I1 showed the highest difference with +14.61 pp, which just missed significance between sports groups (*p* = 0.063). I2 and I3 still showed high differences. In the last three intervals, the differences nearly leveled off with a mean difference of only 1.70 ± 0.11 pp.

**FIGURE 8:**
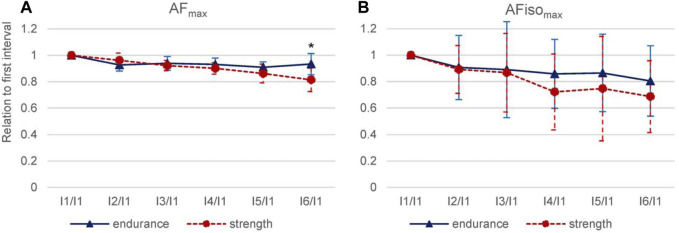
Interval relations comparing strength and endurance athletes. For AF_max_ **(A)** and AFiso_max_ **(B)**, the value of I1 was set at 100%, and the subsequent interval values (I2–I6) were related to I1. M and SD (error bars) are shown for endurance (blue, *n* = 6) and strength (red, *n* = 6) athletes. Significant differences between strength and endurance athletes are indicated by *p = 0.017.

Those findings were supported by considering the relation of each interval to the values of the first interval ( $\frac{{{AF}_{\max}\_I}_{i}}{{AF}_{\max}\_I1}$ or $\frac{{A{Fiso}_{\max}\_I}_{i}}{{A{Fiso}_{\max}\_I}_{1}}$ , i = 1–6) (Figure 8). This showed a stronger decline in strength vs. endurance athletes toward the end of the 30 trials. AF_max_ was significant in a mixed ANOVA regarding intervals*sports (F_G_ (1.980, 19.799) = 4.998, *p* = 0.018, f = 0.707). Pairwise comparisons revealed that the last ratio I6 to I1 was significantly lower for strength vs. endurance athletes (t (10) = 2.442, *p* = 0.017, g = 1.301). For AFiso_max_, a clear separation was visible from I4/I1 to I6/I1, where strength athletes showed lower values. However, the results do not differ significantly between endurance athletes and strength athletes (F (5, 50) = 0.423, *p* = 0.831), presumably due to the high variation.

## 4 Discussion

The main objective of this study was to investigate the behavior of AF parameters in elbow flexors during 30 intermittent repeated trials. As a side consideration, the investigation of maximal torque parameters, especially HIMA vs. PIMA, and a first comparison of strength vs. endurance athletes were conducted. Since AF is still unknown and rarely considered in science, a major part of the discussion should focus on the specialty of this force capacity.

### 4.1 Decline of torque parameters over the course of and after 30 repeated AF trials

According to the hypotheses, the different torque parameters showed a decline over the course of 30 trials with very large effect sizes (g > 1.16). MVIC pre/post and AF_max_ start/end showed a similar decline in torque of around −12% and −13%, respectively. AFiso_max_ declined significantly stronger by around −25% from start to end, which was assumed. A decline in torque after eccentric actions can be the result of central/neurological fatigue (spinal or supraspinal) (Chapman et al., 2005; Prasartwuth et al., 2005; Dartnall et al., 2009; Place et al., 2009; Westerblad et al., 2010), intramuscular processes (Place et al., 2009; Westerblad et al., 2010), metabolic changes (Place et al., 2009; Westerblad et al., 2010), structural exhaustion (Chapman et al., 2005; Prasartwuth et al., 2005; Dartnall et al., 2009), muscle damage (Warren et al., 1999; Nosaka et al., 2001; 2002), mental fatigue, or the lack of motivation (Warren et al., 1999; Dech et al., 2021). Due to the long duration of measurements, the latter two may have occurred. The general exhaustion of participants increased over the course of 30 trials, which is interpreted as normal considering the high number of measurements. However, since the decline in MVIC was according to other investigations with a lower number of measurements (see below), possible effects of mental fatigue or lack of motivation are considered as minor. Furthermore, the different behaviors of the decline regarding MVIC and AF parameters speak against such effects.

The isometric–eccentric muscle action performed in the present investigation refers to muscle lengthening under strain. Thus, proprioceptive signals of Golgi tendon organs (GTOs) must have been present. Possible nociceptive signals from force-transmitting structures (muscle, insertion area, and tendon) could have occurred. Those might have resulted in neurogenic reflective inhibition and, therefore, a decline in force with repeated trials. The “body’s self-regulatory mechanisms of the GTOs in order to protect structures” are known as autogenic inhibition (Hindle et al., 2012). Due to the inhibitory signals of the GTO, the excitability of a contracting or stretched muscle is reduced (Sharman et al., 2006; Hindle et al., 2012). Furthermore, fascia is characterized by a “dense sensory innervation making it highly sensitive to stress and strain” (Satkunskiene et al., 2022). Hence, possible inhibitory effects of fascia (including intramuscular connective tissue) during muscle lengthening under strain might also have been present.

Regarding AF_max_ and MVIC, the reasons for the decline cannot be specified based on the performed investigation. Other parameters such as electromyography (EMG) (Prasartwuth et al., 2005; Dartnall et al., 2009; Place et al., 2009) (especially for investigating fatiguing effects) and blood levels of myofiber proteins or soreness—both parameters for eccentric-induced muscle damage (Warren et al., 1999; Prasartwuth et al., 2005)—could have given more insights. However, the latter two are discussed critically in relation to eccentric-induced muscle injuries, where MVIC torque is seen as the most suitable parameter (Warren et al., 1999). Nosaka et al. found a graduated decline in MVIC after eccentric exercise. Immediately after two maximal eccentric actions (15 s resting period), the MVIC was reduced by 20%, after six eccentric actions by 33%, and after 24 eccentric actions by 56% (Nosaka et al., 2001). Chapman et al. found a decline of ∼10%, however, in a clearly different procedure (eight eccentric actions; MVIC test prior to each eccentric trial; resting period of 60–120 s) (Chapman et al., 2005). Our procedure resembled that of Chapman et al. rather than that of Nosoka et al. because of the longer resting periods. However, we performed a considerably higher number of trials (30x AF vs. 8x eccentric). The decline in MVIC pre vs. post repetition trials by −12.35% ± 8.9% is in accordance with the findings of Chapman et al. Surprisingly, the decline did not turn out to be higher, which would have been conceivable given the higher number of trials. Motivational aspects as reasons for the decline are assumed to be unlikely, since AF_max_ and AFiso_max_ behaved differently. In particular, the stronger decline in AFiso_max_ than in AF_max_ or in MVIC could speak for eccentric-induced muscle damage or the mentioned neurogenic reflective inhibition, which might have had a stronger impairing effect on the more sensitive holding capacity. However, at least partly fatiguing effects are assumed as reasons for the decline, probably paired with eccentric-induced muscle damage, reflective inhibition, or structural exhaustion.

### 4.2 Behavior of torque parameters comparing endurance vs. strength athletes

It is commonly known that strength athletes achieve greater forces than endurance athletes. This was supported by the present findings of MVICpre and AF_max_ at start and for MVICpost, which were significantly lower in endurance vs. strength athletes. AF_max_ at end of 30 trials missed significance (*p* = 0.081). However, AFiso_max_ did not differ significantly between sports groups neither at the start nor at the end, which was not expected. The reason for this might be the high variation of AFiso_max_ or its particularities (see below).

Little seems to be known about the decline after repeated muscle actions or exercise when comparing both sports. The preliminary findings of the present study indicated a non-significant difference between sports groups regarding the amount of percentage decline in maximal torques from start to end. The non-significant results are probably due to the small sample size and considerably high standard deviations in the groups, especially for AFiso_max_. Nevertheless, it seems worth mentioning that strength vs. endurance athletes reduced their torques clearly stronger in regard of AF_max_ start/end (−19% ± 9% vs. −7% ± 8%) and AFiso_max_ start/end (−32% ± 20% vs. −25% ± 30%). For MVIC, however, strength athletes showed a lower torque reduction than endurance athletes (−9% ± 10% vs. −12% ± 7%), which was not expected. The decrease over the course of 30 measurements (regression line and six intervals) was significantly steeper for strength vs. endurance athletes. Moreover, differences regarding the behavior of torque decline appeared between both sports groups. For endurance athletes, the main decline in AF_max_ occurred after the first interval. Thereafter, the torques remained on a similar level and even increased partly. In contrast, strength athletes showed a rather continuous decline from interval to interval. For AF_max_, the first interval was significantly higher than the subsequent intervals. In particular, the pattern of decline in AFiso_max_ was conspicuous in strength athletes, which showed clear decreases after I1 (−11%) and I3 (−15%) and a kind of plateau formation for I2 and I3 and I4–I6 (Figure 6). This is similar to previous findings (Ryan et al., 2021), where a decrease of torques was found, which plateaued in sets 4 and 5 (see Introduction). Endurance athletes showed no such plateau formation. Evidently, AF and MVIC reacted differently to repeated trials considering endurance and strength athletes. Moreover, it was not expected that a stronger reduction in MVIC would be found for endurance vs. strength athletes. This seems to be a contradiction to the stronger decline of AF parameters in strength athletes compared to endurance athletes.

It is known that fast twitch (FT) muscle fibers are less resistant against muscle fatigue than slow twitch (ST) muscle fibers (Zierath and Hawley, 2004; Westerblad et al., 2010; Lievens et al., 2020). If strength athletes had more FT fibers than endurance athletes with a higher number of ST muscle fibers, the stronger decline of AF parameters would have been explainable, but not the lower decrease of MVIC. In strength athletes, the accumulation of, e.g., lactate in the intracellular space, Ca^2+^ myosin ATPase, and Mg^2+^ actomyosin ATPase are presumably higher (Zierath and Hawley, 2004). However, acidosis was not considered to be the crucial factor for fatigue (Westerblad et al., 2002; 2010); rather, the phosphocreatine breakdown resulting in an increase of inorganic phosphate ions which, in turn, has “multiple negative effects on the contractile function of skeletal muscle” (Westerblad et al., 2010). Fatigue after intermittent muscle actions at high intensities (>30% MVC) is assumed to be explained largely by the failure in excitation–contraction coupling (Place et al., 2009). It should be noted that during AF, isometric and eccentric actions occur. It is known that the cross-bridge activity is reduced and central activities are higher during eccentric actions compared to concentric and isometric actions (Bigland and Lippold, 1954; Morgan, 1990; Fang et al., 2001; 2004; Christou et al., 2003; Baudry et al., 2007; Howatson et al., 2011; Duchateau and Enoka, 2016; Barrué-Belou et al., 2018; Valadão et al., 2018). Therefore, regarding AF trials, a neuromuscular fatigue might be assumable, which could have been more intense in strength athletes than in endurance athletes. For the findings regarding the MVIC, the post-activation potentiation (PAP) or the residual force enhancement (rFE) could be relevant. Considering PAP, it was suggested that despite fatigue, voluntary force production might be facilitated by the muscle’s contractile history (Cuenca-Fernández et al., 2017; Blazevich and Babault, 2019). PAP leads to an inhibition of fatigue and is known to enhance muscular activation. Up to 3–4 min post-activation, PAP is known to be strong. Since the MVIC measurements after AF trials were performed after a resting period of ∼2 min, a PAP effect might have been present. According to Boullosa et al., power athletes benefit more from brief maximal conditioning activities than endurance athletes (Boullosa et al., 2018). Hence, this could probably explain the lower decline in MVIC found in strength athletes than in endurance athletes.

rFE was observed because of the greater stiffness of titin when an active muscle is stretched (Herzog et al., 2016). The higher amount of myofilaments in strength athletes could lead to a higher residual stretch resistance compared to endurance athletes. This might explain the lower decline in strength athletes regarding MVIC. However, if a slight effect of PAP or rFE would still be present after 30 maximal holding/decelerating actions with a resting period of 120 s and whether it might have prevented a stronger decline in MVIC in strength athletes cannot be judged based on the current investigation. Furthermore, it should be noted that none of the studies mentioned previously investigated the behavior over the course of or after a series of specific AF actions. Thus, the comparability is limited and speculative. Another explanation could be that due to the higher torques in strength athletes, a reflective neurogenic inhibition might have had a stronger effect in strength vs. endurance athletes, which might be especially relevant for AF parameters.

Nevertheless, MVIC and AF showed different patterns for strength vs. endurance athletes, despite the preliminary character. This can be interpreted as a further reason why AF—especially AFiso_max_—has to be clearly distinguished from commonly assessed strengths. The findings suggest executing further research on the differences between strength and endurance athletes regarding AF. The maximal holding capacity should be considered with regard to exercise or sports performance in strength and endurance athletes, especially concerning risks of injury and the development of musculoskeletal complaints (see below).

### 4.3 Integration of AF parameters into the current concept of strength

AF_max_, as assessed in the present study using the pneumatic measuring system, corresponds to the maximal value after the transition from isometric to eccentric muscle action. It was previously suggested that AF_max_ can be integrated into the conditional abilities of strength (Dech et al., 2021) as MVIC or the maximal eccentric strength measured by isokinetic systems in the sense that AF_max_ reflects maximal force capacities. In the presented study, the maximal torque of AF_max_ of elbow flexors amounted to ∼83% of MVIC. In Dech et al., AF_max_ of elbow extensors amounted to ∼93% of MVIC (Dech et al., 2021). The lower AF_max_ values might result from several factors. First, the AF trials started after at least three MVIC trials were performed. Hence, possible fatiguing effects might have occurred. This aspect is rather unlikely, since even more MVIC tests were performed by Dech et al., but the relation of AF_max_ to MVIC was higher than that in the current study. Moreover, the resting periods can be considered sufficiently long. It seems like the ratio of AF_max_ to MVIC remains similar, irrespective of whether it is gained at the start (∼83%) or end (∼81%) of the repeated trials. Second, the differences between MVIC and AF_max_ might have resulted from the different assessments. For AF_max_, the external load was increased by the pneumatic system so that the individual’s isometric holding force was exceeded and the participant merged into eccentric actions over the course of one trial. Therefore, the duration until AF_max_ was reached was considerably longer (∼11.92 ± 2.12 s) compared to MVIC (∼3.36 ± 1.00 s). Additionally, a combination of isometric and eccentric muscle action was present. There are inconsistent findings on maximal forces/torques comparing isometrics and eccentrics. Some investigations reported higher forces during eccentric vs. isometric actions (Doss and Karpovich, 1965; Seliger et al., 1980; Griffin, 1987; Chapman et al., 2005), but some did not (Westing et al., 1988; 1990; Babault et al., 2001). Duchateau and Enoka postulated that the “rate of change in muscle length must be similar when comparing” shortening and lengthening actions, since the movement velocity influences the neuromuscular activation (Duchateau and Enoka, 2011; 2016). During MVIC, no movement occurred due to the stable abutment. During AF_max_, the velocity depended on the individual braking speed. As a consequence, despite the identical starting position, the muscle lengths differed in reaching MVIC or AF_max_, respectively. Therefore, the comparison is clearly based on different conditions regarding duration, muscle length, and contraction velocity, which might have influenced the maximal torques. However, Dech et al. found very high determination coefficients for MVIC and AF_max_ (*r* ^2^ = 94.09%–96.04%), indicating their strong connection (Dech et al., 2021). The clearly lower determination coefficient regarding MVIC and AFiso_max_ (*r* ^2^ = 72.25%–94.09%) suggested that AFiso_max_ cannot be sufficiently explained by MVIC (Dech et al., 2021).

### 4.4 Holding vs. pushing isometric muscle action: Neuromuscular considerations and particularities of the holding capacity

The highest value of maximal HIMA (AFiso_max_) amounted to 67.67% ± 13.60% of maximal PIMA (MVIC) (ratio of HIMA to PIMA). Hence, maximal HIMA was clearly and significantly lower than maximal PIMA with a very high effect size of g = 1.907. For both, muscle length and time to maximal torque were similar. The duration until the maximal value was reached lasted about 3.36 ± 1.00 s during PIMA (MVIC) and was slightly but not significantly longer for HIMA (AFiso_max_) with 3.98 ± 0.90 s. Therefore, PIMA and HIMA were based on similar conditions; the only difference in execution was that during MVIC, the participants pushed against a stable resistance, and during AFiso_max_, they had to react to the impacting increasing load in a holding manner. In the study by Dech et al., this relation was averagely 84% for elbow extensors. The even lower relation in the present study might be due to reasons such as different muscles (elbow extensors vs. flexors), different adjustments of the measuring system, or different participants. Another influencing factor might be the evaluation of AFiso_max_ data, which is not trivial (see Limitations). Nevertheless, different maximal torques occurred either by pushing or by holding isometrically. This further supports a distinction between two types of isometric muscle action. Presumably, different neuronal control mechanisms are necessary for both types, as discussed in detail previously (Schaefer and Bittmann, 2017; Schaefer and Bittmann, 2021; Schaefer and Bittmann, 2023; Bittmann et al., 2020; Dech et al., 2021; Schaefer et al., 2021a; Schaefer et al., 2021b; Schaefer et al., 2022b). As mentioned above, the muscle action *per se* is isometric in both types. However, by executing MVIC, the participant is acting (pushing). An adjustment of muscle length is not required due to the stable abutment. The sensorimotor component of reacting to an impacting load is not required as for holding actions, especially if the external load is increasing as with AF. For AFiso_max_, the neuromuscular system has to adjust muscle force and length adequately to maintain the isometric position. Therefore, it can be assumed that the neuromuscular control processes are considerably more complex for HIMA than PIMA. A first case study regarding this topic evaluated the brain activity during both types of isometric action of elbow extensors in a setting in which two participants interacted with coupled forearms (one performed PIMA; the other, HIMA; and *vice versa*) (Schaefer and Bittmann, 2023). The brain activity of the left and central areas of the holding partner showed higher coherence with the muscular oscillations of the pushing partner than *vice versa*. It was assumed that HIMA requires a higher level of sensorimotor control than PIMA (Schaefer and Bittmann, 2023). Taken together, the following findings (in addition to others) of several studies support the suggestion of two different types of isometric muscle action: 1) HIMA seems to be more difficult to maintain than PIMA (Hunter et al., 2002; Rudroff et al., 2007; 2011; Schaefer and Bittmann, 2017; Schaefer and Bittmann, 2021); 2) maximal force values during HIMA (AFiso_max_) using the pneumatic measuring system are significantly lower than those during PIMA (MVIC) as shown in this study and in Dech et al. (2021); and 3) the inter-muscle–brain coherence differ significantly between HIMA and PIMA (Schaefer and Bittmann, 2023). We propose to clearly differentiate two types of isometric muscle action and start to implement those findings in sports and movement sciences and in sports medicine. Moreover, studies investigating the holding capacity during MMT showed that AFiso_max_ seems to be vulnerable to impairing inputs already in healthy participants, as, e.g., regarding unpleasant imaginations/odors that are related to the negative emotion of disgust (Schaefer et al., 2021a; Schaefer et al., 2021b; Schaefer et al., 2022b). AFiso_max_ was reduced instantaneously to ∼56% of AF_max_. During positive inputs, AFiso_max_ increased immediately to ∼99% of AF_max_ in the objectified MMT. This instant reaction indicates that the holding capacity can be affected *via* reflex pathways. Therefore, the reduction of AFiso_max_ could be a parameter to assess the functionality of the neuromuscular system.

From a theoretical point of view, we assume that a reduced holding capacity results in an impaired stability of joints during holding or decelerating actions. This might favor injuries or the development of complaints of the musculoskeletal system. It is known that the majority of injuries in competitive sports arise without direct contact and occur during actions including running, twisting, turning, and landing (Read et al., 2016)—all of them involve decelerating and/or holding actions. Based on video analysis, anterior cruciate ligament (ACL) injuries in soccer players occurred in >85% of cases without direct contact to the knee (Waldén et al., 2015; Della Villa et al., 2020). Similarly, in basketball, 72% of ACL injuries did not involve any contact with another player (Krosshaug et al., 2007). These findings are supported by other investigations (Arendt and Dick, 1995; Boden et al., 2000; Johnston et al., 2018). Read et al. proposed that neuromuscular imbalances (strength, coordination/control between lower extremities, or the like) might be crucial risk factors for injuries (Myer et al., 2004; Read et al., 2016). Nevertheless, they stated that “the presence of these deficiencies does not indicate an explicit causative factor for injury *per se*” (Read et al., 2016). An impaired holding capacity might be the key to understanding and investigating injury mechanisms. Van Hooren and Bosch hypothesized that “instability of the fascicle to remain isometric” could lead to injury (Van Hooren and Bosch, 2018). Similarly, as with passive structures, muscle strain injuries also arise during loaded muscle lengthening (Garrett, 1990; 1996; Fridén and Lieber, 2001; Kieb et al., 2010). During concentric contractions, they do not occur. Notably, injuries—irrespective of whether passive or active structures are affected—occur if external loads have to be absorbed. The AF exactly captures this capability. It seems to be important to consider at what force the muscle starts to lengthen if an external load is impacting. The holding capacity (AFiso_max_) must be regarded in relation to the individual’s maximal strength (e.g., MVIC and AF_max_). When a knee rotator releases at 30% of its actual strength, a knee injury can evidently occur if the athlete is turning with their foot fixed in the grass. If muscles are able to maintain an isometric position—and, therefore, stability of the related joints—up to a considerably high amount of the individual’s maximal strength despite an external load, injuries might be prevented. Since the holding capacity apparently seems to be very sensitive to inputs that enter the control circuitries of the neuromuscular system (as, e.g., emotions (Schaefer et al., 2021a; Schaefer et al., 2021b; Schaefer et al., 2022b) or nociceptions (Bittmann, 2021)), the maximal holding capacity is assumed to be a factor with respect to injury risks. Furthermore, it could be an explanation for the still-not-understood appearance of complaints without structural degeneration or even the development of structural degeneration. It is common knowledge that, e.g., low back pain does not correlate with degenerative changes of the spine (Jensen et al., 1994; Rahyussalim et al., 2020). Moreover, asymptomatic individuals show degenerative changes (Brinjikji et al., 2015). Hence, there must be other factors leading to such pain syndromes. We assume that those arise under load if the holding capacity is impaired due to factors such as mental stress, nociception, or the like. Also, in patients with post-infectious syndromes such as long COVID, the holding capacity was found to be significantly reduced, which could explain muscle weakness and musculoskeletal pain in those patients (Schaefer and Bittmann, 2022) (case report under review, conference abstract in Sweeney et al., 2022).

The higher decrease in AFiso_max_ after repetition trials compared to AF_max_ indicates that in healthy participants the holding capacity is stronger reduced after repeated maximal isometric–eccentric muscle activations compared to other strengths. This might support the finding that injuries mostly occur at the end of the half of a match (Price et al., 2004; Read et al., 2016). Considering the higher decrease of AFiso_max_ in the course of fatigue (probably due to the aforementioned factors such as eccentric muscle damage and neurogenic reflective inhibition) in combination with its high variability, single movements with extremely low holding strength can occur. Those circumstances could be seen as risk factors for injuries.

Summarizing these points, the holding capacity seems to occupy a special role in the functionality of the neuromuscular system. Because of its unique behavior, it should be considered regarding injuries and the development of musculoskeletal pain or symptoms such as muscle weakness. The commonly assessed MVIC might not uncover those impairments due to the assumed less complex neuromuscular control and regulation processes. Furthermore, the strength assessment in the sense of AF is closer to motions in real life and sports, since the individual is not forced into particular movements but has to react and adapt to the external load. This is also a benefit compared to the more commonly assessed strengths.

### 4.5 Limitations

The assessment of AF is not trivial. Pneumatics enables the requirements of maintaining an isometric position despite the pressure increase due to the compressibility of air. However, the dynamics of pressure increase change with the expansion of the bellows cylinder; the pressure increase slows down if the bellows cylinder expands. Hence, as the participant merged into eccentric action, the pressure rise would decelerate. That is why partly long durations occurred until AF_max_ was reached. Next generations of the system should first enable a smooth onset and then an exponential increase of pressure rise, passing into a rather linear increase as was proposed for the force increase during the MMT (Bittmann et al., 2020).

Furthermore, the evaluation of the breaking point (maximal holding capacity) is not trivial. AFiso_max_ is not shown by a peak value in the curve but occurs during the force rise. The decisive factor is the moment at which the stable isometric position is left and muscle lengthening occurs. The freely movable limb, the known commonly appearing alterations during isometric muscle action, the probable smooth transitions from isometrics to eccentrics, and the individual differences in decelerating velocity (therefore, partly very flat declines in angles) lead to difficulties in determining this value. However, the algorithm was repeatedly revised based on a large number of measurements (>800), and the current state captures the force at the breaking point accurately and sophisticatedly (Dech et al., 2021). Therefore, we assume that the algorithm contributes—if at all—only minimally to possible incorrect results regarding AFiso_max_ or its high variation. Again, this might be a consequence of the aforementioned specialties of the holding capacity.

Regarding the comparison of types of sports, the small sample size (each *n* = 6) must be mentioned. Despite this, a number of significant differences occurred. However, the results can only be interpreted as preliminary. Furthermore, measuring professional athletes might have had advantages over the non-professional athletes included here, especially regarding the assumptions on muscle fiber types. In professional athletes, the chances are higher that muscle fiber types are more distinct than in non-professional athletes.

The high number of statistical comparisons must also be mentioned as a limitation. Those were accepted according to several authors (O’Brien, 1983; Rothman, 1990; Bender et al., 2002).

## 5 Conclusion

This basic research study approved a significant decline in torque parameters over the course of 30 intermittent repetitions of AF. Based on the chosen setting, the behaviors of AF_max_ and MVIC were similar, with a decline of −13% and −12%, respectively, which can be considered moderate after 30 maximal contractions. In contrast, AFiso_max_ clearly declined by −29% which underpins the particularity of this special holding function. This is presumably based on complex control and regulation processes during such adaptive holding actions. Fatiguing effects, minor eccentric-induced muscle damages, or a neurogenic reflective inhibition were discussed as possible underlying mechanisms but cannot be clarified based on the findings of this study. Further investigations could include further methods for verification.

Based on present and previous findings on HIMA vs. PIMA, the suggestion of two types of isometric muscle action was supported. A distinction between the two types appears to be indicated and should be implemented in sports, movement, and training sciences in education and practice.

Because muscle stability is discussed as a protective factor regarding injury mechanisms, we suggest putting a higher focus on AF with special attention to the holding capacity in further research regarding sports, movement, training, and health science, and in prevention, therapy, and rehabilitation.

Strength and endurance athletes showed different behaviors regarding those specific repetition trials. The results must be regarded as preliminary, not just because of the small sample size. Further investigations are needed.

## Data Availability

The original contributions presented in the study are included in the article/Supplementary Material, further inquiries can be directed to the corresponding author.

## References

[B1] Arendt E. Dick R. (1995). Knee injury patterns among men and women in collegiate basketball and soccer. NCAA data and review of literature. Am. J. Sports Med. 23, 694–701. 10.1177/036354659502300611 8600737

[B2] Babault N. Pousson M. Ballay Y. Van Hoecke J. (2001). Activation of human quadriceps femoris during isometric, concentric, and eccentric contractions. J. Appl. Physiol. 91, 2628–2634. 10.1152/jappl.2001.91.6.2628 11717228

[B3] Baltes-Götz B. (2020). Analyse von hierarchischen linearen Modellen mit SPSS. Available at: https://www.uni-trier.de/fileadmin/urt/doku/hlm/hlm.pdf [Accessed November 3, 2022].

[B4] Barrué-Belou S. Marque P. Duclay J. (2018). Recurrent inhibition is higher in eccentric compared to isometric and concentric maximal voluntary contractions. Acta Physiol 223, e13064. 10.1111/apha.13064 29575639

[B5] Baudry S. Klass M. Pasquet B. Duchateau J. (2007). Age-related fatigability of the ankle dorsiflexor muscles during concentric and eccentric contractions. Eur. J. Appl. Physiol. 100, 515–525. 10.1007/s00421-006-0206-9 16718508

[B6] Bender R. Lange S. Ziegler A. (2002). Multiples Testen - - Artikel Nr. 12 der Statistik-Serie in der DMW - -. DMW - Dtsch. Med. Wochenschr. 127, T 4–T 7. 10.1055/s-2002-32816,

[B7] Bigland B. Lippold O. C. J. (1954). The relation between force, velocity and integrated electrical activity in human muscles. J. Physiol. 123, 214–224. 10.1113/jphysiol.1954.sp005044 13131257 PMC1366165

[B8] Bittmann F. (2021). Adaptive force: a novel concept of neuromuscular function. Padua. Available at: https://www.youtube.com/watch?v=U9Z5H3Fifv4.

[B9] Bittmann F. N. Dech S. Aehle M. Schaefer L. V. (2020). Manual Muscle Testing—Force Profiles and Their Reproducibility. Diagnostics 10, 996. 10.3390/diagnostics10120996 33255648 PMC7759939

[B10] Blazevich A. J. Babault N. (2019). Post-activation Potentiation Versus Post-activation Performance Enhancement in Humans: Historical Perspective, Underlying Mechanisms, and Current Issues. Front. Physiol. 10, 1359. 10.3389/fphys.2019.01359 31736781 PMC6838751

[B11] Boden B. P. Dean G. S. Feagin J. A. Garrett W. E. (2000). Mechanisms of Anterior Cruciate Ligament Injury. Orthopedics 23, 573–578. 10.3928/0147-7447-20000601-15 10875418

[B12] Boullosa D. Del Rosso S. Behm D. G. Foster C. (2018). Post-activation potentiation (PAP) in endurance sports: A review. Eur. J. Sport Sci. 18, 595–610. 10.1080/17461391.2018.1438519 29490594

[B13] Brinjikji W. Luetmer P. H. Comstock B. Bresnahan B. W. Chen L. E. Deyo R. A. (2015). Systematic Literature Review of Imaging Features of Spinal Degeneration in Asymptomatic Populations. Am. J. Neuroradiol. 36, 811–816. 10.3174/ajnr.A4173 25430861 PMC4464797

[B14] Chapman D. Newton M. Nosaka K. (2005). Eccentric torque-velocity relationship of the elbow flexors. Isokinet. Exerc. Sci. 13, 139–145. 10.3233/IES-2005-0192

[B15] Christou E. A. Shinohara M. Enoka R. M. (2003). Fluctuations in acceleration during voluntary contractions lead to greater impairment of movement accuracy in old adults. J. Appl. Physiol. 95, 373–384. 10.1152/japplphysiol.00060.2003 12651861

[B16] Clarkson P. M. Kroll W. McBride T. C. (1980). Maximal isometric strength and fiber type composition in power and endurance athletes. Eur. J. Appl. Physiol. 44, 35–42. 10.1007/BF00421761 7190494

[B17] Cohen J. (1988). Statistical power analysis for the behavioral sciences. 2nd ed. Hillsdale, N.J: L. Erlbaum Associates.

[B18] Cohen J. (1992). A power primer. Psychol. Bull. 112, 155–159. 10.1037/0033-2909.112.1.155 19565683

[B19] Conable K. M. Rosner A. L. (2011). A narrative review of manual muscle testing and implications for muscle testing research. J. Chiropr. Med. 10, 157–165. 10.1016/j.jcm.2011.04.001 22014904 PMC3259988

[B20] Cuenca-Fernández F. Smith I. C. Jordan M. J. MacIntosh B. R. López-Contreras G. Arellano R. (2017). Nonlocalized postactivation performance enhancement (PAPE) effects in trained athletes: a pilot study. Appl. Physiol. Nutr. Metab. 42, 1122–1125. 10.1139/apnm-2017-0217 28675792

[B21] Dartnall T. J. Rogasch N. C. Nordstrom M. A. Semmler J. G. (2009). Eccentric Muscle Damage Has Variable Effects on Motor Unit Recruitment Thresholds and Discharge Patterns in Elbow Flexor Muscles. J. Neurophysiol. 102, 413–423. 10.1152/jn.91285.2008 19420118

[B22] de Paula Simola R. Á. Raeder C. Wiewelhove T. Kellmann M. Meyer T. Pfeiffer M. (2016). Muscle mechanical properties of strength and endurance athletes and changes after one week of intensive training. J. Electromyogr. Kinesiol. 30, 73–80. 10.1016/j.jelekin.2016.05.005 27317976

[B23] Dech S. Bittmann F. N. Schaefer L. V. (2021). Assessment of the Adaptive Force of Elbow Extensors in Healthy Subjects Quantified by a Novel Pneumatically Driven Measurement System with Considerations of Its Quality Criteria. Diagnostics 11, 923. 10.3390/diagnostics11060923 34063869 PMC8224031

[B24] Della Villa F. Buckthorpe M. Grassi A. Nabiuzzi A. Tosarelli F. Zaffagnini S. (2020). Systematic video analysis of ACL injuries in professional male football (soccer): injury mechanisms, situational patterns and biomechanics study on 134 consecutive cases. Br. J. Sports Med. 54, 1423–1432. 10.1136/bjsports-2019-101247 32561515

[B25] Doss W. S. Karpovich P. V. (1965). A comparison of concentric, eccentric, and isometric strength of elbow flexors. J. Appl. Physiol. 20, 351–353. 10.1152/jappl.1965.20.2.351

[B26] Duchateau J. Enoka R. M. (2011). Human motor unit recordings: Origins and insight into the integrated motor system. Brain Res 1409, 42–61. 10.1016/j.brainres.2011.06.011 21762884

[B27] Duchateau J. Enoka R. M. (2016). Neural control of lengthening contractions. J. Exp. Biol. 219, 197–204. 10.1242/jeb.123158 26792331

[B28] Fang Y. Siemionow V. Sahgal V. Xiong F. Yue G. H. (2001). Greater Movement-Related Cortical Potential During Human Eccentric Versus Concentric Muscle Contractions. J. Neurophysiol. 86, 1764–1772. 10.1152/jn.2001.86.4.1764 11600637

[B29] Fang Y. Siemionow V. Sahgal V. Xiong F. Yue G. H. (2004). Distinct brain activation patterns for human maximal voluntary eccentric and concentric muscle actions. Brain Res 1023, 200–212. 10.1016/j.brainres.2004.07.035 15374746

[B30] Fridén J. Lieber R. L. (2001). Eccentric exercise-induced injuries to contractile and cytoskeletal muscle fibre components: Exercise-induced muscle injury. Acta Physiol. Scand. 171, 321–326. 10.1046/j.1365-201x.2001.00834.x 11412144

[B31] Gacesa J. Z. P. Klasnja A. V. Grujic N. G. (2013). Changes in Strength, Endurance, and Fatigue During a Resistance-Training Program for the Triceps Brachii Muscle. J. Athl. Train. 48, 804–809. 10.4085/1062-6050-48.4.16 23914910 PMC3867092

[B32] Garrett W. E. (1990). Muscle strain injuries: clinical and basic aspects. Med. Sci. Sports Exerc. 22, 436–443. 10.1249/00005768-199008000-00003 2205779

[B33] Garrett W. E. (1996). Muscle Strain Injuries. Am. J. Sports Med. 24, S2–S8. 10.1177/036354659602406S02 8947416

[B34] Glowacki S. P. Martin S. E. Maurer A. Baek W. Green J. S. Crouse S. F. (2004). Effects of Resistance, Endurance, and Concurrent Exercise on Training Outcomes in Men. Med. Sci. Sports Exerc. 36, 2119–2127. 10.1249/01.MSS.0000147629.74832.52 15570149

[B35] Griffin J. W. (1987). Differences in Elbow Flexion Torque Measured Concentrically, Eccentrically, and Isometrically. Phys. Ther. 67, 1205–1208. 10.1093/ptj/67.8.1205 3615588

[B36] Häkkinen K. Keskinen K. L. (1989). Muscle cross-sectional area and voluntary force production characteristics in elite strength- and endurance-trained athletes and sprinters. Eur. J. Appl. Physiol. 59, 215–220. 10.1007/BF02386190 2583165

[B37] Herzog W. Schappacher G. DuVall M. Leonard T. R. Herzog J. A. (2016). Residual Force Enhancement Following Eccentric Contractions: A New Mechanism Involving Titin. Physiology 31, 300–312. 10.1152/physiol.00049.2014 27252165

[B38] Hindle K. Whitcomb T. Briggs W. Hong J. (2012). Proprioceptive Neuromuscular Facilitation (PNF): Its Mechanisms and Effects on Range of Motion and Muscular Function. J. Hum. Kinet. 31, 105–113. 10.2478/v10078-012-0011-y 23487249 PMC3588663

[B39] Hoff M. Schaefer L. Heinke N. Bittmann F. (2015). Report on Adaptive Force, a specific neuromuscular function. Eur. J. Transl. Myol. 25, 5183. 10.4081/ejtm.2015.5183 26913155 PMC4748997

[B40] Howatson G. Taylor M. B. Rider P. Motawar B. R. McNally M. P. Solnik S. (2011). Ipsilateral motor cortical responses to TMS during lengthening and shortening of the contralateral wrist flexors: Contraction-specificity in the ipsilateral M1. Eur. J. Neurosci. 33, 978–990. 10.1111/j.1460-9568.2010.07567.x 21219480 PMC3075420

[B41] Hunter S. K. Ryan D. L. Ortega J. D. Enoka R. M. (2002). Task Differences With the Same Load Torque Alter the Endurance Time of Submaximal Fatiguing Contractions in Humans. J. Neurophysiol. 88, 3087–3096. 10.1152/jn.00232.2002 12466432

[B42] Jensen M. C. Brant-Zawadzki M. N. Obuchowski N. Modic M. T. Malkasian D. Ross J. S. (1994). Magnetic Resonance Imaging of the Lumbar Spine in People without Back Pain. N. Engl. J. Med. 331, 69–73. 10.1056/NEJM199407143310201 8208267

[B43] Johnston J. T. Mandelbaum B. R. Schub D. Rodeo S. A. Matava M. J. Silvers-Granelli H. J. (2018). Video Analysis of Anterior Cruciate Ligament Tears in Professional American Football Athletes. Am. J. Sports Med. 46, 862–868. 10.1177/0363546518756328 29466019

[B44] Kieb M. Lorbach O. Engelhardt M. (2010). Muscle injuries: diagnostics and treatments. Orthop 39, 1098–1107. 10.1007/s00132-010-1693-2 21103858

[B45] Krosshaug T. Nakamae A. Boden B. P. Engebretsen L. Smith G. Slauterbeck J. R. (2007). Mechanisms of anterior cruciate ligament injury in basketball: video analysis of 39 cases. Am. J. Sports Med. 35, 359–367. 10.1177/0363546506293899 17092928

[B46] Lievens E. Klass M. Bex T. Derave W. (2020). Muscle fiber typology substantially influences time to recover from high-intensity exercise. J. Appl. Physiol. 128, 648–659. 10.1152/japplphysiol.00636.2019 31999527

[B47] Mileva K. N. Morgan J. Bowtell J. (2009). Differentiation of power and endurance athletes based on their muscle fatigability assessed by new spectral electromyographic indices. J. Sports Sci. 27, 611–623. 10.1080/02640410802707011 19296362

[B48] Morgan D. L. (1990). New insights into the behavior of muscle during active lengthening. Biophys. J. 57, 209–221. 10.1016/S0006-3495(90)82524-8 2317547 PMC1280663

[B49] Myer G. D. Ford K. R. Hewett T. E. (2004). Rationale and Clinical Techniques for Anterior Cruciate Ligament Injury Prevention Among Female Athletes. J. Athl. Train. 39, 352–364. 15592608 PMC535528

[B50] Nosaka K. Newton M. Sacco P. (2002). Delayed-onset muscle soreness does not reflect the magnitude of eccentric exercise-induced muscle damage: DOMS and muscle damage. Scand. J. Med. Sci. Sports 12, 337–346. 10.1034/j.1600-0838.2002.10178.x 12453160

[B51] Nosaka K. Sakamoto K. Newton M. Sacco P. (2001). The repeated bout effect of reduced-load eccentric exercise on elbow flexor muscle damage. Eur. J. Appl. Physiol. 85, 34–40. 10.1007/s004210100430 11513318

[B52] O’Brien P. C. (1983). The appropriateness of analysis of variance and multiple-comparison procedures. Biometrics 39, 787–794. 10.2307/2531110 6652209

[B53] Oranchuk D. J. Diewald S. N. McGrath J. W. Nelson A. R. Storey A. G. Cronin J. B. (2021a). Kinetic and kinematic profile of eccentric quasi-isometric loading. Sports Biomech, 1–14. 10.1080/14763141.2021.1890198 33666143

[B54] Oranchuk D. J. Nelson A. R. Storey A. G. Diewald S. N. Cronin J. B. (2021b). Short-term neuromuscular, morphological, and architectural responses to eccentric quasi-isometric muscle actions. Eur. J. Appl. Physiol. 121, 141–158. 10.1007/s00421-020-04512-4 32995961

[B55] Oranchuk D. J. Storey A. G. Nelson A. R. Cronin J. B. (2019). Scientific Basis for Eccentric Quasi-Isometric Resistance Training: A Narrative Review. J. Strength Cond. Res. 33, 2846–2859. 10.1519/JSC.0000000000003291 31361732

[B56] Place N. Bruton J. D. Westerblad H. (2009). MECHANISMS OF FATIGUE INDUCED BY ISOMETRIC CONTRACTIONS IN EXERCISING HUMANS AND IN MOUSE ISOLATED SINGLE MUSCLE FIBRES. Clin. Exp. Pharmacol. Physiol. 36, 334–339. 10.1111/j.1440-1681.2008.05021.x 18671711

[B57] Prasartwuth O. Taylor J. L. Gandevia S. C. (2005). Maximal force, voluntary activation and muscle soreness after eccentric damage to human elbow flexor muscles: Eccentric damage and muscle activation. J. Physiol. 567, 337–348. 10.1113/jphysiol.2005.087767 15946963 PMC1474152

[B58] Price R. J. Hawkins R. D. Hulse M. A. Hodson A. (2004). The Football Association medical research programme: an audit of injuries in academy youth football. Br. J. Sports Med. 38, 466–471. 10.1136/bjsm.2003.005165 15273188 PMC1724880

[B59] Rahyussalim A. J. Zufar M. L. L. Kurniawati T. (2020). Significance of the Association between Disc Degeneration Changes on Imaging and Low Back Pain: A Review Article. Asian Spine J 14, 245–257. 10.31616/asj.2019.0046 31679325 PMC7113468

[B60] Read P. J. Oliver J. L. De Ste Croix M. B. A. Myer G. D. Lloyd R. S. (2016). Neuromuscular Risk Factors for Knee and Ankle Ligament Injuries in Male Youth Soccer Players. Sports Med 46, 1059–1066. 10.1007/s40279-016-0479-z 26856339 PMC5501175

[B61] Rothman K. J. (1990). No Adjustments Are Needed for Multiple Comparisons. Epidemiology 1, 43–46. 10.1097/00001648-199001000-00010 2081237

[B62] Rudroff T. Barry B. K. Stone A. L. Barry C. J. Enoka R. M. (2007). Accessory muscle activity contributes to the variation in time to task failure for different arm postures and loads. J. Appl. Physiol. 102, 1000–1006. 10.1152/japplphysiol.00564.2006 17095642

[B63] Rudroff T. Justice J. N. Holmes M. R. Matthews S. D. Enoka R. M. (2011). Muscle activity and time to task failure differ with load compliance and target force for elbow flexor muscles. J. Appl. Physiol. 110, 125–136. 10.1152/japplphysiol.00605.2010 21030676 PMC3253001

[B64] Rudroff T. Kalliokoski K. K. Block D. E. Gould J. R. Klingensmith W. C. I. Enoka R. M. (2013). PET/CT imaging of age- and task-associated differences in muscle activity during fatiguing contractions. J. Appl. Physiol. 114, 1211–1219. 10.1152/japplphysiol.01439.2012 23412899 PMC3656430

[B65] Ryan E. D. Gerstner G. R. Mota J. A. Trexler E. T. Giuliani H. K. Blue M. N. M. (2021). The Acute Effects of a Multi-Ingredient Herbal Supplement on Performance Fatigability: A Double-Blind, Randomized, and Placebo-Controlled Trial. J. Diet. Suppl. 18, 507–516. 10.1080/19390211.2020.1790709 32723193

[B66] Satkunskiene D. Ardekani M. M. Z. Khair R. M. Kutraite G. Venckuniene K. Snieckus A. (2022). Warm-Up and Hamstrings Stiffness, Stress Relaxation, Flexibility, and Knee Proprioception in Young Soccer Players. J. Athl. Train. 57, 485–493. 10.4085/1062-6050-0416.20 34185855 PMC9205552

[B67] Schaefer L. Hoff M. Bittmann F. (2017). Measuring system and method of determining the Adaptive Force. Eur. J. Transl. Myol. 27, 6479. 10.4081/ejtm.2017.6479 29118954 PMC5656809

[B68] Schaefer L. V. Bittmann F. N. (2017). Are there two forms of isometric muscle action? Results of the experimental study support a distinction between a holding and a pushing isometric muscle function. BMC Sports Sci. Med. Rehabil. 9, 11. 10.1186/s13102-017-0075-z 28503309 PMC5426061

[B69] Schaefer L. V. Bittmann F. N. (2019). Muscular Pre-activation Can Boost the Maximal Explosive Eccentric Adaptive Force. Front. Physiol. 10, 910. 10.3389/fphys.2019.00910 31396096 PMC6663982

[B70] Schaefer L. V. Bittmann F. N. (2021). Paired personal interaction reveals objective differences between pushing and holding isometric muscle action. PLOS ONE 16, e0238331. 10.1371/journal.pone.0238331 33956801 PMC8101915

[B73] Schaefer L. V. Dech S. Aehle M. Bittmann F. N. (2021a). Disgusting odours affect the characteristics of the Adaptive Force in contrast to neutral and pleasant odours. Sci. Rep. 11, 16410. 10.1038/s41598-021-95759-0 34385522 PMC8361115

[B74] Schaefer L. V. Dech S. Bittmann F. N. (2021b). Adaptive Force and emotionally related imaginations – preliminary results suggest a reduction of the maximal holding capacity as reaction to disgusting food imagination. Heliyon 7, e07827. 10.1016/j.heliyon.2021.e07827 34485726 PMC8391030

[B72] Schaefer L. V. Bittmann F. N. (2022). The Adaptive Force as potential biomechanical parameter in the recovery process of patients with long COVID, 2022110400. Preprint. 10.20944/preprints202211.0400.v1 PMC10000769 36900026

[B75] Schaefer L. V. Dech S. Wolff L. L. Bittmann F. N. (2022a). Emotional Imagery Influences the Adaptive Force in Young Women: Unpleasant Imagery Reduces Instantaneously the Muscular Holding Capacity. Brain Sci 12, 1318. 10.3390/brainsci12101318 36291257 PMC9599475

[B76] Schaefer L. V. Dech S. Wolff L. L. Bittmann F. N. (2022b). Influence of emotionally affective imaginations on the Adaptive Force in young women: unpleasant imaginations reduce the holding capacity of muscles. In Review. 10.21203/rs.3.rs-1281031/v3

[B71] Schaefer L. V. Bittmann F. N. (2023). Case report: Individualized pulsed electromagnetic field therapy in a Long COVID patient using the Adaptive Force as biomarker. Front. Med. 9, 879971. 10.3389/fmed.2022.879971 PMC9874300 36714125

[B77] Seliger V. Dolejš L. Karas V. (1980). A dynamometric comparison of maximum eccentric, concentric, and isometric contractions using EMG and energy expenditure measurements. Eur. J. Appl. Physiol. 45, 235–244. 10.1007/BF00421331 7193132

[B78] Sharman M. J. Cresswell A. G. Riek S. (2006). Proprioceptive Neuromuscular Facilitation Stretching: Mechanisms and Clinical Implications. Sports Med 36, 929–939. 10.2165/00007256-200636110-00002 17052131

[B79] Small K. McNaughton L. Greig M. Lovell R. (2009). Effect of Timing of Eccentric Hamstring Strengthening Exercises During Soccer Training: Implications for Muscle Fatigability. J. Strength Cond. Res. 23, 1077–1083. 10.1519/JSC.0b013e318194df5c 19528859

[B80] Smith L. L. Fulmer M. G. Holbert D. McCammon M. R. Houmard J. A. Frazer D. D. (1994). The impact of a repeated bout of eccentric exercise on muscular strength, muscle soreness and creatine kinase. Br. J. Sports Med. 28, 267–271. 10.1136/bjsm.28.4.267 7894959 PMC1332088

[B81] Sullivan G. M. Feinn R. (2012). Using Effect Size—or Why the *P* Value Is Not Enough. J. Grad. Med. Educ. 4, 279–282. 10.4300/JGME-D-12-00156.1 23997866 PMC3444174

[B82] Sweeney H. L. Masiero S. Carraro U. (2022). The 2022 On-site Padua Days on Muscle and Mobility Medicine hosts the University of Florida Institute of Myology and the Wellstone Center, March 30 - April 3, 2022 at the University of Padua and Thermae of Euganean Hills, Padua, Italy: The collection of abstracts. Eur. J. Transl. Myol. 32, 10440. 10.4081/ejtm.2022.10440 35272451 PMC8992680

[B83] Valadão P. Kurokawa S. Finni T. Avela J. (2018). Effects of muscle action type on corticospinal excitability and triceps surae muscle-tendon mechanics. J. Neurophysiol. 119, 563–572. 10.1152/jn.00079.2017 29118191

[B84] Van Hooren B. Bosch F. (2018). Preventing hamstring injuries – Part 2: There is possibly an isometric action of the hamstrings in high-speed running and it does matter. Sports Perform. Sci. Rep. v1. Available at: https://sportperfsci.com/wp-content/uploads/2018/04/SPSR28_HS-serie_Bas-Bosch_180418_final.pdf.

[B85] Waldén M. Krosshaug T. Bjørneboe J. Andersen T. E. Faul O. Hägglund M. (2015). Three distinct mechanisms predominate in non-contact anterior cruciate ligament injuries in male professional football players: a systematic video analysis of 39 cases. Br. J. Sports Med. 49, 1452–1460. 10.1136/bjsports-2014-094573 25907183 PMC4680158

[B86] Warren G. L. Lowe D. A. Armstrong R. B. (1999). Measurement tools used in the study of eccentric contraction-induced injury. Sports Med 27, 43–59. 10.2165/00007256-199927010-00004 10028132

[B87] Westerblad H. Allen D. G. Lännergren J. (2002). Muscle Fatigue: Lactic Acid or Inorganic Phosphate the Major Cause?. Physiology 17, 17–21. 10.1152/physiologyonline.2002.17.1.17 11821531

[B88] Westerblad H. Bruton J. D. Katz A. (2010). Skeletal muscle: Energy metabolism, fiber types, fatigue and adaptability. Exp. Cell Res. 316, 3093–3099. 10.1016/j.yexcr.2010.05.019 20580710

[B89] Westing S. H. Seger J. Y. Karlson E. Ekblom B. (1988). Eccentric and concentric torque-velocity characteristics of the quadriceps femoris in man. Eur. J. Appl. Physiol. 58, 100–104. 10.1007/BF00636611 3203653

[B90] Westing S. H. Seger J. Y. Thorstensson A. (1990). Effects of electrical stimulation on eccentric and concentric torque-velocity relationships during knee extension in man. Acta Physiol. Scand. 140, 17–22. 10.1111/j.1748-1716.1990.tb08971.x 2275401

[B91] Zierath J. R. Hawley J. A. (2004). Skeletal Muscle Fiber Type: Influence on Contractile and Metabolic Properties. PLoS Biol 2, e348. 10.1371/journal.pbio.0020348 15486583 PMC521732

