# Supplementary material for: Repeated measurements of Adaptive Force: Maximal holding capacity differs from other maximal strength parameters and preliminary characteristics for non-professional strength vs. endurance athletes
Source: Front Physiol. 2023 Feb 22;14:1020954. doi: 10.3389/fphys.2023.1020954 (PMC9992808; doi:10.3389/fphys.2023.1020954)
Supplement: Supplementary file 1 [file DataSheet1.PDF]

frontiers

Supplementary Material

Contents

Supplementary Table 1. Technical specifications of the pneumatic AF measuring system 2
Supplementary Table 2. Maximal voluntary isometric contraction. 3
Supplementary Table 3. Maximal Adaptive Force...4
Supplementary Table 4. Maximal isometric Adaptive Force..5

Supplementary Table 1. Technical specifications of the pneumatic AF measuring system.

System components (company)	Specification
Compressor<br>(JUN-AIR International A/S, Nørresundby, Denmark)	Model 6, Serial-No. 700367; Condor MDR2 EN 60947-4-1;<br>max. system pressure: 8 bar,<br>adjusted to max. 2 bar for the present investigation
Bellows cylinder<br>(Zitec, Plattling, Germany)	Zitec SP-2 B04,; 2-fold, Ø 165 mm, max force: 9 kN,<br>stroke length: 1–110 mm (adjustable), rise time: 0.1–30 s continuously
Control unit<br>(Seifert Drucklufttechnik GmbH, Bernsbach, Germany)	Pressure reduction to max. 1 bar
Pressure sensor in control unit<br>(Seifert Drucklufttechnik GmbH, Bernsbach, Germany)	Linear 1 V = 1.05 bar
Motor for throttle control<br>(RS Components GmbH, Frankfurt/Main, Germany)	RS PRO High Torque, DC Geared Motor (24 V dc, 13.2 W, 0.75 Ncm, 300 rpm)
Strain gauge and amplifier<br>(modified by Biovision, Wehrheim, Germany)	LMZ 2000N 3006, linearly, 1 V = 195.082 N
Acceleration sensors (ACC)<br>(modified by co. Biovision, Wehrheim, Germany)	Sensitivity 312 mV/g (range ± 2g) cosinusoidal,<br>between 70–110° approx. linear, linearity: ± 0.2%
A/D converter<br>(National Instruments, modified by Biovision, Wehrheim, Germany)	16-bit, range: -5 to 5 V

Supplementary Table 2. Maximal voluntary isometric contraction. Single values of all trials (M1 to M3 before and M1 to M2 after the 30 repeated AF trials) of the maximal voluntary isometric contraction (MVIC) of each participant (endurance: 1 to 6; strength: 7 to 12). The arithmetic means (M), standard deviations (SD), coefficient of variation (CV) and maximum of each participant as well as regarding all participants, endurance and strength athletes are given.

		**MVIC before 30 AF trials**							**MVIC after the 30 AF trials**					
Participants		M1	M2	M3	M	SD	CV	max	M1	M2	M	SD	CV	max
endurance athletes	1	71.70	74.88	75.80	74.13	2.15	0.03	75.80	74.61	73.41	74.01	0.85	0.01	74.61
	2	60.37	66.53	65.13	64.01	3.23	0.05	66.53	50.63	51.45	51.04	0.58	0.01	51.45
	3	62.89	62.13	63.08	62.70	0.50	0.01	63.08	51.28	54.46	52.87	2.25	0.04	54.46
	4	79.33	76.66	74.76	76.92	2.30	0.03	79.33	68.92	67.60	68.26	0.94	0.01	68.92
	5	91.76	90.09	87.14	89.66	2.34	0.03	91.76	71.40	70.47	70.93	0.66	0.01	71.40
	6	73.52	75.88	79.76	76.39	3.15	0.04	79.76	62.78	61.22	62.00	1.11	0.02	62.78
Strength athletes	7	75.48	76.87	74.83	75.73	1.04	0.01	76.87	68.83	78.22	73.53	6.64	0.09	78.22
	8	102.53	101.26	100.36	101.38	1.09	0.01	102.53	82.66	81.99	82.33	0.47	0.01	82.66
	9	92.86	100.47	99.18	97.51	4.07	0.04	100.47	86.56	85.93	86.25	0.44	0.01	86.56
	10	116.85	112.79	109.83	113.16	3.53	0.03	116.85	93.47	96.61	95.04	2.22	0.02	96.61
	11	86.44	90.26	93.54	90.08	3.55	0.04	93.54	89.95	91.07	90.51	0.79	0.01	91.07
	12	58.89	65.93	66.17	63.67	4.14	0.06	66.17	60.81	64.69	62.75	2.74	0.04	64.69
total n = 12	M	81.05	82.81	82.47	82.11	2.59	0.03	84.39	71.82	73.09	72.46	1.64	0.02	73.62
	SD	17.72	16.01	15.39	16.24	1.22	0.02	16.68	14.24	14.18	14.12	1.76	0.02	14.10
	CV	0.22	0.19	0.19	0.20	0.47	0.52	0.20	0.20	0.19	0.19	1.07	1.05	0.19
endurance athletes	M	73.26	74.36	74.28	73.97	2.28	0.03	76.04	63.27	63.10	63.19	1.06	0.02	63.94
	SD	11.45	9.65	9.02	9.87	0.98	0.01	10.30	10.30	8.88	9.57	0.61	0.01	9.39
	CV	0.16	0.13	0.12	0.13	0.43	0.47	0.14	0.16	0.14	0.15	0.58	0.71	0.15
strength athletes	M	88.84	91.26	90.65	90.25	2.90	0.03	92.74	80.38	83.09	81.73	2.22	0.03	83.30
	SD	20.34	17.31	16.69	17.99	1.45	0.02	18.41	12.83	11.12	11.86	2.37	0.03	11.14
	CV	0.23	0.19	0.18	0.20	0.50	0.60	0.20	0.16	0.13	0.15	1.07	1.13	0.13

Supplementary Table 3. Maximal Adaptive Force. Single values of the maximal Adaptive Force (AF_{max}) of each participant (endurance 1 to 6) and strength (7 to 12) of each of the 30 AF trials (M1 to M30). The arithmetic means (M), standard deviations (SD) and coefficient of variation (CV) of each trial regarding all participants, endurance and strength athletes as well as for each participant regarding the 30 trials are given.

trial	endurance athletes						strength athletes						total n = 12			endurance			strength		
	1	2	3	4	5	6	1	2	3	4	5	6	M	SD	CV	M	SD	CV	M	SD	CV
M1	56.65	66.43	55.05	55.80	64.06	69.03	67.51	90.31	60.44	95.11	72.04	67.56	68.33	12.68	0.19	61.17	6.08	0.10	75.50	13.93	0.18
M2	53.48	60.68	55.85	58.53	65.16	60.57	65.16	86.20	59.85	91.36	71.94	66.54	66.28	11.67	0.18	59.04	4.09	0.07	73.51	12.54	0.17
M3	45.10	59.94	52.19	51.61	64.80	61.51	62.83	81.58	58.15	91.02	69.22	65.77	63.64	12.71	0.20	55.86	7.43	0.13	71.43	12.44	0.17
M4	39.38	59.75	52.50	53.29	55.81	60.34	62.15	85.74	66.88	94.62	70.25	64.37	63.76	14.81	0.23	53.51	7.64	0.14	74.00	13.12	0.18
M5	41.96	57.57	53.21	50.22	63.58	56.81	66.05	83.18	68.98	86.75	71.61	65.59	63.79	13.00	0.20	53.89	7.38	0.14	73.69	9.07	0.12
M6	39.49	58.32	54.92	44.04	60.31	57.29	62.59	81.29	61.47	84.88	79.23	68.55	62.70	13.97	0.22	52.39	8.53	0.16	73.00	10.10	0.14
M7	41.93	57.07	52.48	42.26	61.98	53.24	65.53	80.97	55.15	84.38	69.78	64.15	60.74	13.32	0.22	51.49	8.02	0.16	70.00	10.97	0.16
M8	46.11	55.30	52.24	43.93	52.13	60.65	63.75	81.41	55.77	87.16	76.70	63.77	61.58	13.77	0.22	51.73	6.09	0.12	71.43	12.15	0.17
M9	40.69	54.84	55.35	49.92	61.16	61.94	69.06	78.18	54.16	83.76	78.18	62.57	62.48	12.80	0.20	53.98	7.88	0.15	70.98	11.17	0.16
M10	41.22	55.69	53.82	54.29	60.25	56.28	64.80	77.92	54.33	85.46	64.98	61.52	60.88	11.67	0.19	53.59	6.48	0.12	68.17	11.41	0.17
M11	35.13	51.42	54.75	47.64	52.09	57.80	64.23	80.56	54.48	83.75	63.52	64.80	59.18	13.49	0.23	49.81	7.95	0.16	68.55	11.24	0.16
M12	47.85	54.00	56.29	55.07	61.81	56.69	63.37	78.95	64.63	88.44	61.28	63.94	62.69	11.15	0.18	55.29	4.53	0.08	70.10	11.01	0.16
M13	38.32	55.97	55.88	58.52	59.84	55.05	61.85	81.08	52.22	84.10	64.88	59.38	60.59	12.21	0.20	53.93	7.86	0.15	67.25	12.63	0.19
M14	52.59	54.71	50.95	48.38	57.30	56.49	64.56	78.83	60.83	84.92	50.72	60.49	60.06	11.29	0.19	53.40	3.42	0.06	66.73	12.74	0.19
M15	44.30	49.15	54.84	56.13	58.78	54.84	61.14	77.79	49.13	86.96	65.10	60.74	59.91	12.15	0.20	53.01	5.30	0.10	66.81	13.50	0.20
M16	45.60	53.85	53.00	50.01	59.57	54.89	65.59	75.88	52.41	88.56	66.05	61.69	60.59	12.11	0.20	52.82	4.71	0.09	68.36	12.46	0.18
M17	44.68	56.38	51.99	54.98	58.62	54.52	61.20	77.08	50.11	85.93	61.78	58.12	59.62	11.43	0.19	53.53	4.86	0.09	65.70	13.23	0.20
M18	37.38	52.44	55.54	54.56	58.37	58.00	62.23	74.00	56.27	81.71	71.54	56.79	59.90	11.50	0.19	52.72	7.83	0.15	67.09	10.28	0.15
M19	46.07	53.53	51.78	52.82	54.15	55.17	62.28	77.94	48.31	87.17	59.35	59.65	59.02	12.07	0.20	52.25	3.24	0.06	65.78	14.16	0.22
M20	36.18	52.94	52.73	54.97	59.49	56.89	60.32	76.84	53.40	80.43	58.14	61.18	58.63	11.41	0.19	52.20	8.25	0.16	65.05	10.92	0.17
M21	35.59	54.97	52.39	50.12	58.71	59.44	60.58	77.73	48.37	85.54	65.05	61.67	59.18	13.15	0.22	51.87	8.74	0.17	66.49	13.25	0.20
M22	42.49	50.03	50.74	48.94	55.90	56.61	60.59	72.21	42.05	82.40	64.07	60.23	57.19	11.82	0.21	50.79	5.15	0.10	63.59	13.51	0.21
M23	43.84	52.02	52.62	53.54	59.54	53.89	61.49	74.82	42.93	85.37	50.90	55.12	57.17	12.16	0.21	52.58	5.06	0.10	61.77	15.78	0.26
M24	42.59	53.97	51.54	49.22	58.37	55.61	58.37	73.77	46.47	78.73	63.01	57.09	57.40	10.49	0.18	51.88	5.55	0.11	62.91	11.77	0.19
M25	41.57	51.77	52.10	54.81	49.51	52.30	56.55	77.28	47.22	82.58	65.55	55.27	57.21	12.10	0.21	50.34	4.62	0.09	64.07	13.69	0.21
M26	47.19	52.74	48.56	55.98	66.18	53.98	55.65	74.91	41.78	75.83	42.80	54.69	55.86	11.20	0.20	54.10	6.78	0.13	57.61	14.92	0.26
M27	47.35	49.29	53.14	53.77	62.51	53.20	58.93	70.42	42.21	85.67	54.18	52.99	56.97	11.57	0.20	53.21	5.22	0.10	60.73	15.26	0.25
M28	39.66	51.22	51.26	56.84	56.43	52.83	60.22	74.85	44.00	87.39	54.91	58.14	57.31	12.83	0.22	51.37	6.24	0.12	63.25	15.45	0.24
M29	52.20	44.64	56.15	58.07	58.70	53.84	59.80	67.88	40.74	75.80	51.53	47.53	55.57	9.67	0.17	53.93	5.18	0.10	57.21	13.14	0.23
M30	39.22	50.38	52.98	55.10	54.38	52.75	60.45	75.77	44.99	81.96	59.82	54.78	56.88	11.86	0.21	50.80	5.90	0.12	62.96	13.64	0.22
M	43.53	54.37	53.23	52.45	58.98	56.75	62.29	78.18	52.59	85.26	63.94	60.49									
SD	5.38	4.20	1.88	4.31	4.02	3.59	3.03	4.68	7.81	4.49	8.64	4.80									
CV	0.12	0.08	0.04	0.08	0.07	0.06	0.05	0.06	0.15	0.05	0.14	0.08									

Supplementary Table 4. Maximal isometric Adaptive Force. Single values of maximal isometric Adaptive Force ($AFiso_{max}$) of each participant (endurance 1 to 6) and strength (7 to 12) of each of the 30 AF trials (M1 to M30). The arithmetic means (M), standard deviations (SD) and coefficient of variation (CV) of each trial regarding all participants, endurance and strength athletes as well as for each participant regarding the 30 trials are given.

trial	endurance athletes						strength athletes						total n = 12			endurance			strength		
	1	2	3	4	5	6	1	2	3	4	5	6	M	SD	CV	M	SD	CV	M	SD	CV
M1	32.09	43.47	27.57	41.67	51.49	66.65	54.33	60.42	33.63	65.89	47.17	50.22	47.88	12.85	0.27	43.82	14.05	0.32	51.94	11.25	0.22
M2	36.81	36.93	37.10	42.43	41.98	13.37	54.77	64.04	38.35	84.66	58.37	39.34	45.68	17.82	0.39	34.77	10.80	0.31	56.59	17.21	0.30
M3	18.39	31.43	29.55	30.83	33.69	10.13	52.66	59.81	2.07	89.66	33.95	41.00	36.10	23.31	0.65	25.67	9.31	0.36	46.52	29.12	0.63
M4	15.04	28.60	36.20	22.20	44.85	28.21	58.71	77.09	12.90	94.58	38.15	49.49	42.17	24.72	0.59	29.18	10.44	0.36	55.15	28.83	0.52
M5	24.48	28.73	30.96	13.64	47.75	12.95	58.77	66.85	23.77	86.72	38.15	34.99	38.98	22.30	0.57	26.42	12.88	0.49	51.54	23.45	0.45
M6	14.94	34.74	33.19	27.34	16.65	24.21	56.28	32.54	19.96	75.25	25.85	44.54	33.79	17.53	0.52	25.18	8.23	0.33	42.40	20.74	0.49
M7	14.86	26.76	28.96	11.51	48.09	29.10	42.99	27.42	24.26	69.29	37.31	43.49	33.67	15.77	0.47	26.55	12.96	0.49	40.79	16.05	0.39
M8	18.36	25.74	38.23	19.56	36.64	26.92	43.13	70.82	38.68	82.64	50.74	32.09	40.30	19.61	0.49	27.57	8.35	0.30	53.02	19.70	0.37
M9	11.76	26.42	43.84	12.94	41.97	44.66	40.14	61.26	33.87	81.36	30.71	21.52	37.54	19.79	0.53	30.26	15.40	0.51	44.81	22.31	0.50
M10	14.70	23.15	43.47	43.81	53.00	35.49	49.28	58.37	20.63	79.01	37.70	20.76	39.95	18.67	0.47	35.60	14.31	0.40	44.29	22.74	0.51
M11	12.54	27.40	52.20	19.29	6.64	33.07	52.02	33.40	36.61	80.26	27.83	34.58	34.65	19.79	0.57	25.19	16.34	0.65	44.12	19.47	0.44
M12	18.64	18.72	42.69	35.14	42.04	31.52	44.20	72.65	24.43	83.67	20.70	33.63	39.00	20.56	0.53	31.46	10.75	0.34	46.55	26.03	0.56
M13	11.87	20.99	43.46	46.62	31.54	28.25	54.17	62.51	30.32	75.07	36.04	21.52	38.53	18.54	0.48	30.46	13.20	0.43	46.61	20.62	0.44
M14	10.95	17.98	44.93	33.64	37.32	37.97	52.59	66.81	42.64	82.55	21.37	19.10	38.99	21.04	0.54	30.47	13.11	0.43	47.51	25.06	0.53
M15	6.43	19.49	46.09	31.04	25.67	10.41	37.91	18.85	21.77	78.07	27.43	19.03	28.52	19.06	0.67	23.19	14.49	0.63	33.84	22.82	0.67
M16	15.51	31.82	40.01	37.70	18.59	18.46	51.70	38.37	27.51	68.21	33.71	18.80	33.37	15.51	0.46	27.01	10.79	0.40	39.72	17.78	0.45
M17	14.17	23.04	44.77	16.92	52.02	19.32	38.77	36.34	22.60	66.80	34.60	17.04	32.20	16.36	0.51	28.38	15.95	0.56	36.02	17.31	0.48
M18	12.39	16.74	38.97	33.67	26.90	38.30	36.83	33.30	25.71	70.38	37.43	18.07	32.39	15.06	0.46	27.83	11.23	0.40	36.95	17.97	0.49
M19	14.03	15.60	36.73	35.09	32.63	41.06	34.75	11.29	14.07	82.87	43.43	15.25	31.40	20.13	0.64	29.19	11.48	0.39	33.61	27.35	0.81
M20	13.62	22.77	38.27	12.34	33.13	24.51	46.17	4.15	30.37	74.15	40.88	13.57	29.49	19.08	0.65	24.11	10.32	0.43	34.88	25.00	0.72
M21	12.70	21.98	40.58	33.05	34.79	36.21	51.61	9.19	29.76	73.51	24.74	18.70	32.23	17.67	0.55	29.88	10.45	0.35	34.58	23.76	0.69
M22	11.30	21.88	41.26	31.95	35.38	34.06	51.03	10.57	22.49	77.81	37.35	15.83	32.58	18.90	0.58	29.31	10.85	0.37	35.85	25.34	0.71
M23	11.77	20.96	34.87	25.65	34.59	17.74	50.04	6.89	34.31	76.33	23.33	15.30	29.31	19.03	0.65	24.26	9.28	0.38	34.36	25.48	0.74
M24	11.93	16.56	36.75	33.53	43.61	35.37	49.56	49.19	26.70	69.73	30.87	15.05	34.90	16.67	0.48	29.62	12.48	0.42	40.18	19.71	0.49
M25	13.13	18.77	41.64	14.56	35.33	28.64	36.40	12.77	40.46	62.05	37.98	22.18	30.33	14.81	0.49	25.34	11.70	0.46	35.31	16.91	0.48
M26	24.84	16.77	41.89	10.07	54.85	34.38	37.11	59.63	10.45	65.75	13.06	15.11	31.99	20.06	0.63	30.47	16.59	0.54	33.52	24.59	0.73
M27	15.55	18.87	40.23	8.53	44.98	18.50	30.47	59.11	29.48	69.02	31.28	21.10	32.26	18.20	0.56	24.44	14.63	0.60	40.08	19.19	0.48
M28	15.48	12.36	38.87	10.44	34.46	21.38	28.81	55.27	24.11	68.86	18.09	25.19	29.44	17.64	0.60	22.16	11.91	0.54	36.72	20.39	0.56
M29	16.64	12.81	18.67	38.84	47.34	12.18	26.30	20.20	28.75	73.72	27.44	12.55	27.95	18.05	0.65	24.41	14.91	0.61	31.49	21.54	0.68
M30	13.08	19.68	44.13	18.74	43.28	37.11	11.47	41.30	33.45	74.72	25.19	15.34	31.46	18.19	0.58	29.34	13.74	0.47	33.58	23.00	0.68
	15.93	23.37	38.54	26.42	37.71	28.34	44.43	42.68	26.80	76.09	33.03	25.48									
SD	6.27	7.23	6.66	11.62	11.18	12.14	10.98	22.96	9.21	7.79	9.80	11.60									
CV	0.39	0.31	0.17	0.44	0.30	0.43	0.25	0.54	0.34	0.10	0.30	0.46									
